# Insights into the Relationship between Cobamide Synthase and the Cell Membrane

**DOI:** 10.1128/mBio.00215-21

**Published:** 2021-03-23

**Authors:** Victoria L. Jeter, Jorge C. Escalante-Semerena

**Affiliations:** a Department of Microbiology, University of Georgia, Athens, Georgia, USA; University of Washington

**Keywords:** B_12_ biosynthesis, cobamide 5′-phosphate synthase (CobS) enzyme, nucleotide loop assembly, liposome-enhanced CobS activity, vitamin metabolism, membrane-associated metabolism

## Abstract

Cobamides are cobalt-containing cyclic tetrapyrroles used by cells from all domains of life but only produced *de novo* by some bacteria and archaea. The “late steps” of the adenosylcobamide biosynthetic pathway are responsible for the assembly of the nucleotide loop and are required during *de novo* synthesis and precursor salvaging. These steps are characterized by activation of the corrin ring and lower ligand base, condensation of the activated precursors to adenosylcobamide phosphate, and removal of the phosphate, yielding a complete adenosylcobamide molecule. The condensation of the activated corrin ring and lower ligand base is performed by an integral membrane protein, cobamide (5′ phosphate) synthase (CobS), and represents an important convergence of two pathways necessary for nucleotide loop assembly. Interestingly, membrane association of this penultimate step is conserved among all cobamide producers, yet the physiological relevance of this association is not known. Here, we present the purification and biochemical characterization of the CobS enzyme of the enterobacterium Salmonella enterica subsp. *enterica* serovar Typhimurium strain LT2, investigate its association with liposomes, and quantify the effect of the lipid bilayer on its enzymatic activity and substrate affinity. We report a purification scheme that yields pure CobS protein, allowing *in vitro* functional analysis. Additionally, we report a method for liposome reconstitution of CobS, allowing for physiologically relevant studies of this inner membrane protein in a phospholipid bilayer. *In vitro* and *in vivo* data reported here expand our understanding of CobS and the implications of membrane-associated adenosylcobamide biosynthesis.

## Abstract (precis)

*Salmonella* is a human pathogen of worldwide importance, and coenzyme B_12_ is critical for the pathogenic lifestyle of this bacterium. The importance of the work reported here lies on the improvements to the methodology used to isolate cobamide synthase, a polytopic integral membrane protein that catalyzes the penultimate step of coenzyme B_12_ biosynthesis.

## INTRODUCTION

Cobamides (Cbas) are complex cobalt-containing cyclic tetrapyrroles that belong to the family of cofactors known as “the pigments of life,” which includes chlorophylls, coenzyme F_430_, and hemes (1, 2). To date, only some archaea and bacteria are known to make Cbas *de novo*, and although organisms from all domains of life may require Cbas to survive, higher plants are not known to make or use Cbas in their metabolisms (3). Notably, some microalgae use different forms of Cbas (4–7).

Cbas are involved in diverse reactions, including reductive dehalogenation, elimination reactions, enzymatic carbon-skeleton rearrangements, radical *S*-adenosylmethionine (SAM)-catalyzed carbon skeleton rearrangements, and methyl group transfers (8). Recently, Cbas were shown to serve as a light sensor associated with a transcription factor used to regulate gene expression (9).

**FIG 1:**
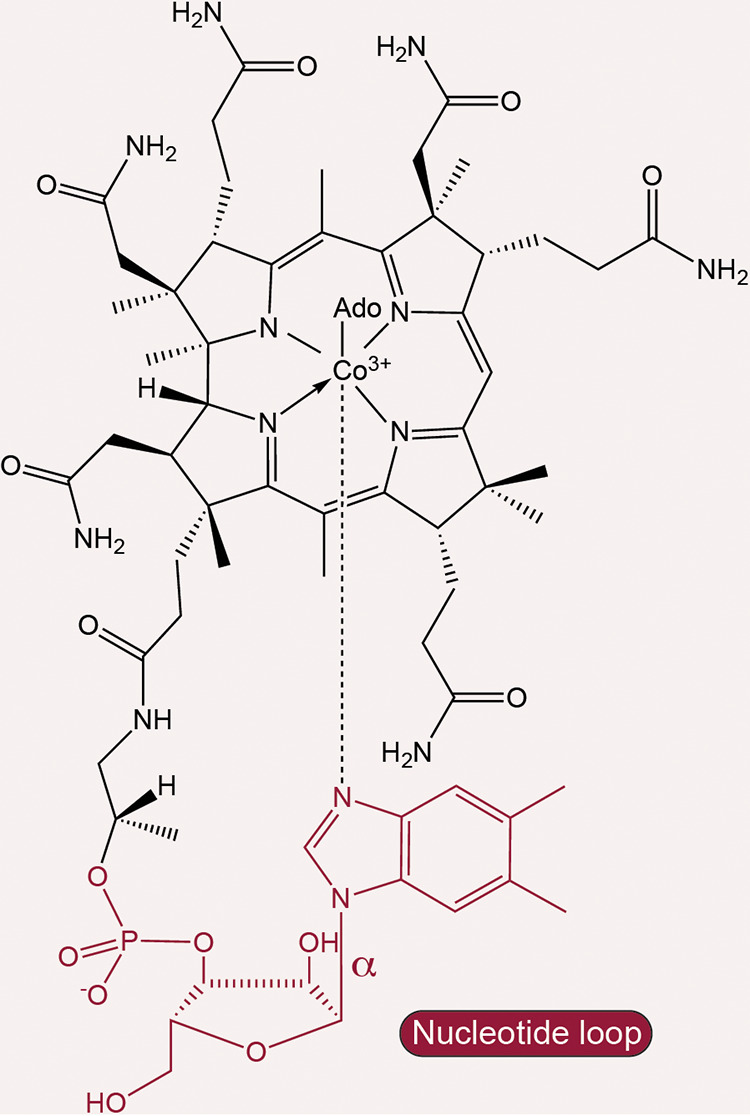
Chemical structure of adenosyl-cobalamin (AdoCbl; coenzyme B_12_).

Cbas are structurally unique among cyclic tetrapyrroles in that they have upper and lower axial ligands. Cobalamin (Cbl) is the cobamide whose structure includes a 5,6-dimethylbenzimidazole (DMB) nucleobase (Fig. 1). The coenzyme form of Cbl is adenosylcobalamin (AdoCbl), which contains a 5′-deoxyadenosine (Ado) as the upper axial ligand. The lower axial ligand is a source of structural diversity among Cbas in that a number of purine and benzimidazole analogues, as well as *p-*cresol and phenol, can serve as the nucleobase (10–12).

Some organisms synthesize AdoCbl *de novo*, yet others rely on salvaging incomplete corrinoids such as cobinamide (Cbi) or cobyric acid (Cby). Salmonella enterica subsp. *enterica* serovar Typhimurium strain LT2 (henceforth, *S.* Typhimurium) synthesizes AdoCbl *de novo* under anoxic conditions, but this bacterium can synthesize Ado-cobamide (AdoCba) under normal oxygen levels in air (i.e., 21% O_2_, also known as normoxic) when incomplete corrinoids such as Cbi or Cby are present in the environment (13, 14).

**FIG 2:**
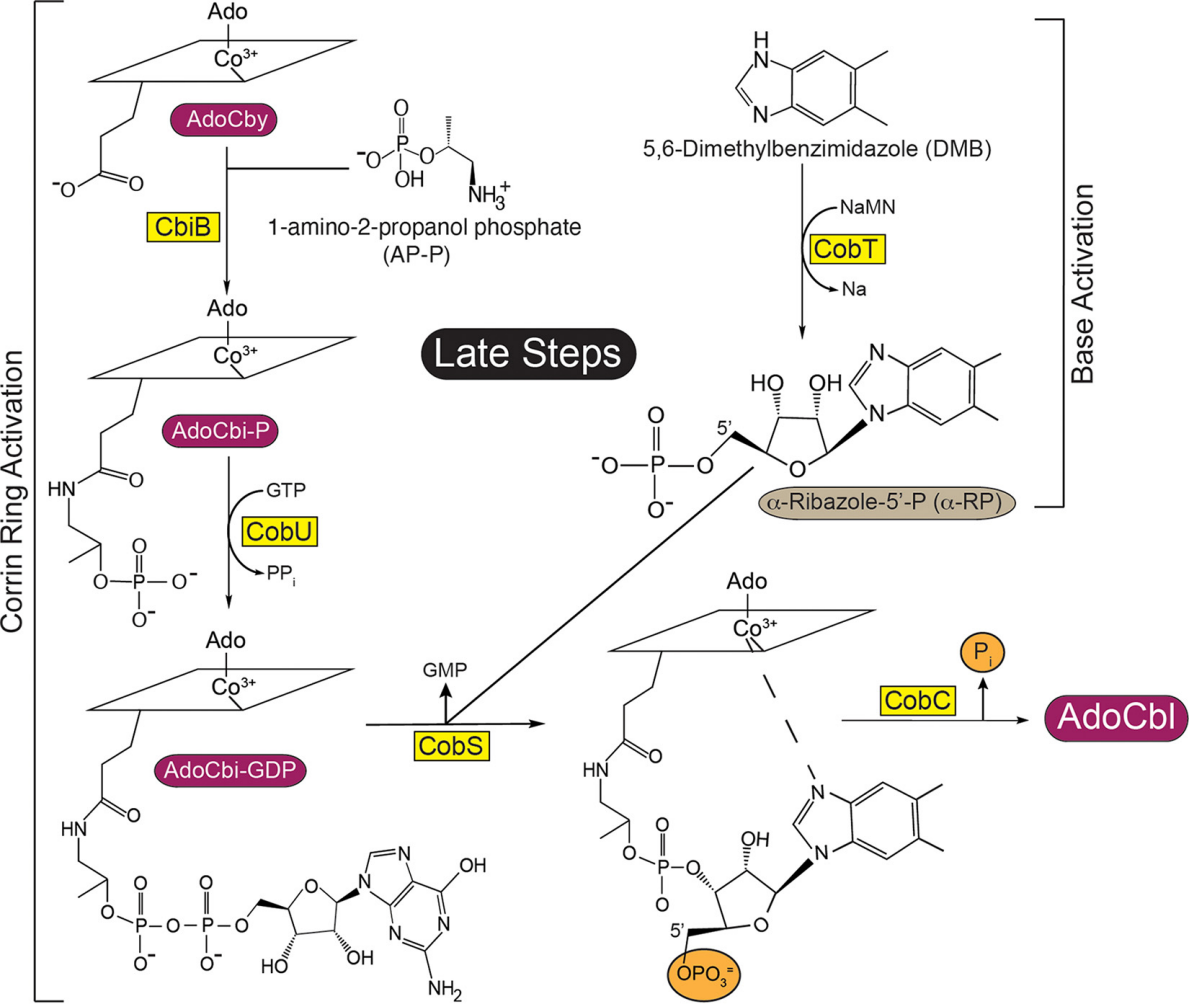
Late steps of Ado-cobamide (AdoCba) biosynthesis in *S.* Typhimurium. AdoCbi (cyclic tetrapyrrole ring) is activated to AdoCbi-GDP by CobU, and DMB (nucleobase) is activated to α-RP by CobT. Cobamide synthase (CobS) catalyzes the penultimate step of cobamide biosynthesis, condensing AdoCbi-GDP and α-RP to yield AdoCbl-P. In the final step, CobC removes the phosphate yielding AdoCbl. NaMN, nicotinic acid mononucleotide.

The late steps of AdoCbl biosynthesis, often referred to as nucleotide loop assembly (NLA) pathway, are responsible for attaching the lower ligand to the corrin ring. In *S.* Typhimurium, the NLA pathway is composed of two branches: (i) ring activation and (ii) base activation (Fig. 2). Ring activation starts with the attachment of 1-amino-propanol phosphate (AP-P) to adenosyl-Cby (AdoCby) to yield adenosylcobinamide phosphate (AdoCbi-P). This reaction is catalyzed by the AdoCbi-P synthase (CbiB, EC 6.3.1.10) enzyme (15). AdoCbi-P is further activated by guanylylation to yield AdoCbi-GDP, a reaction catalyzed by the guanylyltransferase activity of the bifunctional enzyme CobU (NTP:AdoCbi kinase, EC 2.7.7.62; GTP:AdoCbi-P guanylyltransferase, EC 2.7.1.156) (16–18); AdoCbi-GDP is the activated form of the ring. In this bacterium, nucleobase activation is catalyzed by the phosphoribosyltransferase (PRTase) activity of CobT (EC 2.4.2.21). In this reaction, CobT transfers the phosphoribosyl moiety of nicotinate mononucleotide (NaMN) to 5,6-dimethylbenzimidazole (DMB) via an inversion-of-configuration displacement that yields α-ribazole phosphate (α-RP), releasing nicotinic acid (19–23); α-RP is the activated form of the DMB nucleobase. The two active intermediates are the substrates of the cobamide 5′-P synthase (henceforth, cobamide synthase) CobS (EC 2.7.8.26), which condenses AdoCbi-GDP and α-RP to generate adenosylcobalamin 5′ phosphate (henceforth, AdoCbl-P) (24, 25). In the final step, AdoCbl-P is dephosphorylated by the CobC enzyme (EC 3.1.3.73) to yield AdoCbl (26, 27).

**FIG 3:**
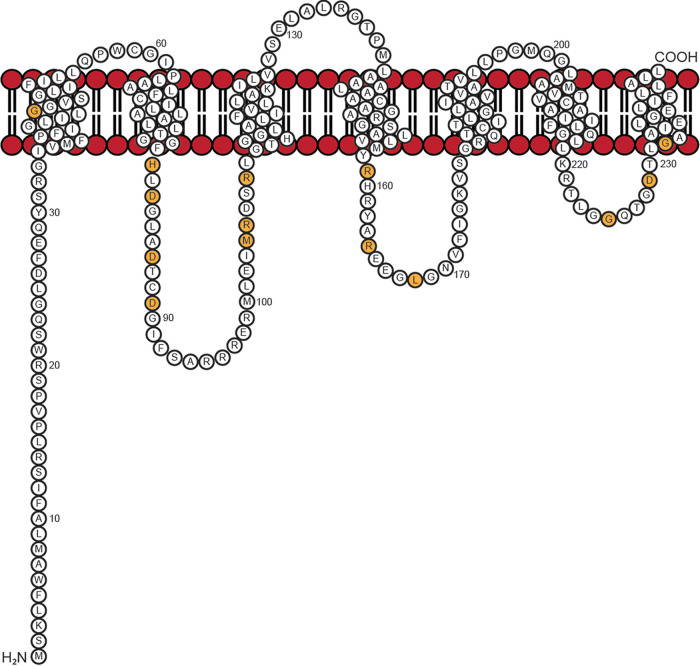
Model of CobS membrane topology. The topology of CobS was previously studied using a series of alkaline phosphatase and β-galactosidase fusions (24). Residues targeted for *in vivo* analysis of variant proteins are indicated in orange.

CobS is a polytopic inner membrane protein that catalyzes the penultimate step of the AdoCbl biosynthesis pathway (Fig. 3) (24). Given that all genomes of cobamide-producing bacteria and archaea sequenced to date contain CobS homologues, we propose that the late steps of cobamide biosynthesis (i.e., the assembly of the nucleotide loop) are catalyzed by a multienzyme complex (i.e., CbiB, CobU, CobT, CobC, and CobS) associated with the cell membrane. Why should the late steps of cobamide biosynthesis localize to cell membranes of bacteria and archaea occupying such diverse environments? This is a question that remains unanswered, leaving an important gap in our understanding of the synthesis of coenzyme B_12_ unfilled.

Our understanding of the cobamide synthase (CobS) function has been hampered by the difficulty in the overproduction and isolation of pure protein. Here, we report several breakthroughs toward a better understanding of the late steps of cobamide biosynthesis: (i) a new and improved protocol for the isolation of the *S.* Typhimurium CobS enzyme that yields 96% homogenous protein; (ii) the reconstitution of purified CobS protein into liposomes to investigate the effect of the lipid bilayer on CobS function, (iii) results of *in vitro* substrate binding analysis, and (iv) results of *in vivo* CobS variant analyses that identify residues and motifs needed for cobamide synthase function.

## RESULTS AND DISCUSSION

### Improved protocol for the isolation of the cobamide 5′-P (CobS) synthase of *S.* Typhimurium.

**FIG 4:**
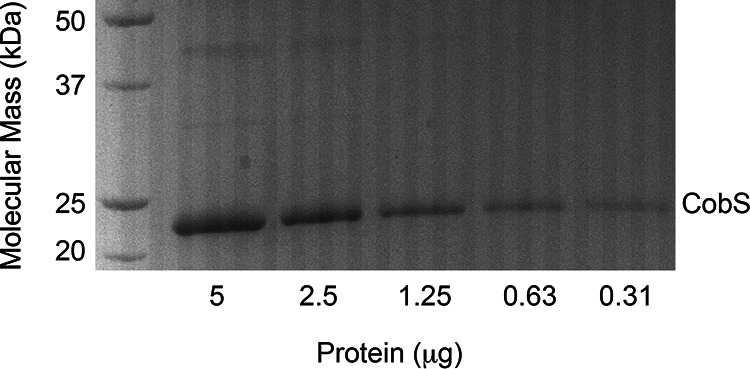
Assessment of CobS purity. SDS-PAGE behavior of lipid-free CobS compared to that of Bio-Rad Precision Plus protein standards. CobS was estimated to be 96% homogenous based on densitometry analysis of the data shown.

The purification of CobS has been problematic (25), with some improvement in yield and purity accomplished over time (24, 27). However, none of those protocols yielded the quality and quantity of protein needed to perform detailed *in vitro* analysis of the function of this key enzyme of the AdoCbl biosynthesis pathway. The new method for the purification of the *S.* Typhimurium CobS enzyme described herein yields protein at 96% homogeneity (Fig. 4), with a yield of 0.5 mg of protein/g cells (wet weight). We utilized phospholipid solubilization of CobS with affinity purification to increase our yield and purity compared to those with previous purification protocols. Attempts to solubilize CobS with octylphenoxypoly(ethyleneoxy)ethanol (Nonidet P-40), Brij35, 3-[(3-cholamidopropyl)dimethylammonio]-1-propanesulfonate hydrate (CHAPS), lauryldimethylamine oxide (LDAO), and *n*-tetradecyl-*N-N*-dimethylglycine detergents were unsuccessful.

### The activity of cobamide synthase increases significantly when it is inserted into a lipid bilayer.

To study the function of CobS in a more physiologically relevant context, we inserted CobS protein into liposomes. The protocol for generating CobS-containing proteoliposomes is detailed in Materials and Methods and Fig. S1.

10.1128/mBio.00215-21.4 FIG S1 Chemical structures of lipids used in this study. The structures of lipids used for liposome formation and CobS solubilization are shown. POPC (1-palmitoyl-2-oleoyl-glycero-3-phosphocholine), POPE (1-palmitoyl-2-oleoyl-*sn*-glycero-3-phosphoethanolamine), POPS (1-palmitoyl-2-oleoyl-*sn*-glycero-3-phoshpo-l-serine), and Rh-DHPE (Lissamine rhodamine B 1,2-dihexadecanoyl-*sn*-glycero-3-phosphoethanolamine) were used to generate liposomes. DHPC (1,2-diheptanoyl-*sn*-glycero-phosphocholine) was used to solubilize CobS during the purification process. Structures were generated using ChemDraw v19.0. Download FIG S1, PDF file, 0.4 MB. Copyright © 2021 Jeter and Escalante-Semerena. 2021 Jeter and Escalante-Semerena. https://creativecommons.org/licenses/by/4.0/ This content is distributed under the terms of the Creative Commons Attribution 4.0 International license.

**FIG 5:**
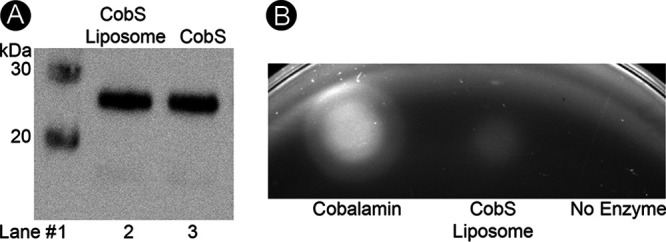
CobS retains activity after insertion into liposomes. (A) Western blot analysis of liposome flotation assay. Purified CobS protein is provided as a control. (B) Bioassay of CobS function reconstituted in liposomes. A 2-μl sample of each reaction mixture was applied to an agar overlay containing strain JE8248. Cells were overlaid on minimal medium containing glycerol as the sole source of carbon and energy. Reactions contained CobS proteoliposomes or liposomes devoid of CobS. Cyanocobalamin was provided as positive control.

The presence of CobS in the phospholipid bilayer of liposomes was confirmed by probing with anti-CobS antibodies (Fig. 5A). Additionally, we probed for activity of CobS when reconstituted in the lipid bilayer using a bioassay. Growth of an S. enterica Δ*cobS* strain embedded in an agar overlay was contingent upon the product of a CobS proteoliposome reaction generated *in vitro* (Fig. 5B). The growth observed in the bioassay confirmed that CobS was functional upon reconstitution in the proteoliposome.

**FIG 6:**
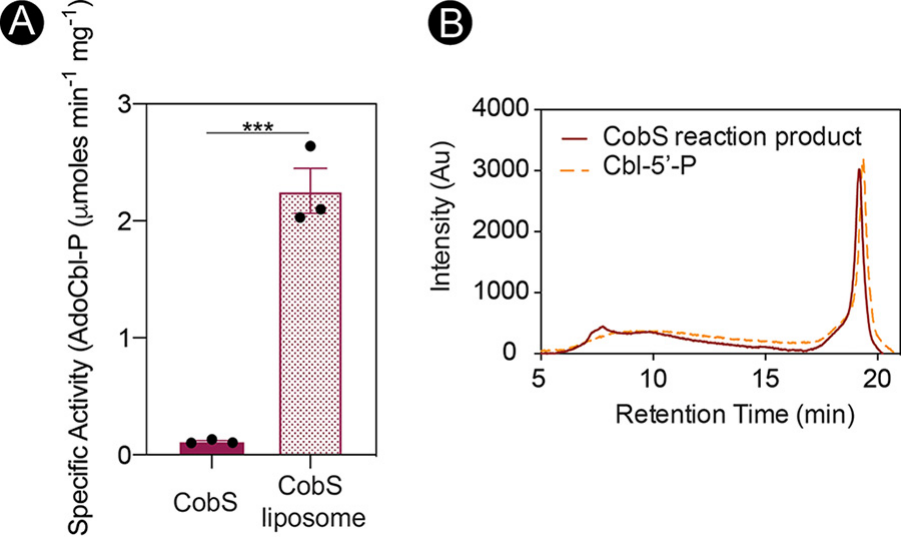
CobS activity substantially increases in liposomes. (A) The specific activities (μmol AdoCbl-P min^−1 ^mg^−1^) of purified CobS and CobS-containing liposomes were determined using a continuous spectrophotometric assay. Error bars represent standard errors of the means (SEMs) from technical triplicates. Asterisks represent significance as determined by unpaired Student's *t* test. ***, *P* < 0.0005. (B) HPLC separations of the reaction product from CobS assay (solid red line) and cobamide phosphate (dashed orange line) are shown.

To quantify the effect of lipids on CobS function, we used the newly implemented continuous spectrophotometric assay described in Materials and Methods. Using this method, we measured a striking difference in specific activity between CobS and CobS embedded into liposomes. That is, the specific activity of CobS was >20-fold lower than that of CobS embedded into liposomes (0.11 versus 2.26 μmol AdoCbl-P min^−1 ^mg^−1^ of protein, respectively) (Fig. 6A). Confirmation of reaction products was determined by reverse-phase high-pressure liquid chromatography (HPLC), using authentic Cbl 5′-P as the positive control (Fig. 6B). Clearly, a lipid environment substantially increased CobS function.

### The protocol used for the generation of CobS proteoliposomes yields near-monodisperse proteoliposomes.

**FIG 7:**
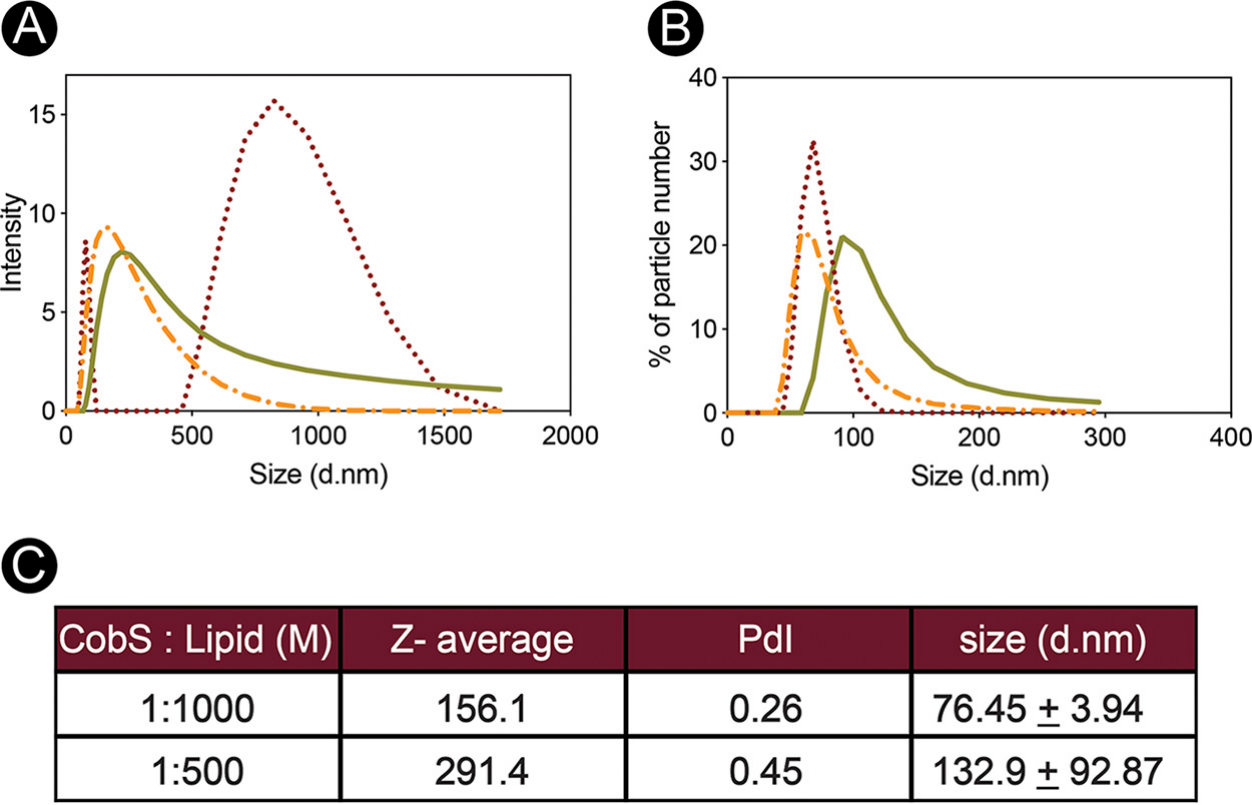
Characteristics of CobS proteoliposome populations. CobS proteoliposome populations were analyzed by dynamic light scattering using a Malvern Zetasizer. Intensity (A) and percentage of particle number (B) measurements over a range of sizes (diameter, nm [d.nm]) are shown for CobS proteoliposomes (1:1,000 lipid ratio, dashed orange trace; 1:500 lipid ratio, solid green trace) and empty liposomes (dotted red trace). (C) Z-average, polydispersity index (PdI), and average size (diameter in nanometers [d.nm]) of both CobS-to-lipid molar ratios tested.

To quantify the activity of CobS in the proteoliposome, we needed to ensure that the population of proteoliposomes was near homogenous. To assess homogeneity, we used dynamic light scattering (DLS) to determine the ideal protein-to-lipid ratio and survey the variance and hydrodynamic properties of CobS-containing proteoliposome populations (Fig. 7). In Fig. 7A, the intensity of distribution of particle sizes is shown for empty liposomes (dotted red trace), CobS-containing proteoliposomes generated with a protein-to-lipid molar ratio of 1:1,000 (dashed orange trace), and CobS-containing proteoliposomes generated with a protein-to-lipid molar ratio of 1:500 (solid green trace). Intensity distribution is a sensitive measure of aggregation, and here we see the most consistent distribution of particle size in the CobS-containing proteoliposomes generated using a 1:1,000 protein/lipid ratio. Figure 7B displays the percent particle number distribution of particle size of the same samples. Again, we observed that CobS-containing proteoliposomes generated with a protein-to-lipid ratio of 1:1,000 showed the least variance in particle size. Figure 7C shows the second-order values determined by dynamic light scattering. Here, we saw that the protein-to-lipid ratio of 1:1,000 yielded a population of proteoliposomes with more consistent Z-average, polydispersity index (PdI), and size. Given the low PdI, lower Z-average, and smaller standard deviation of particle size, we concluded that our population of CobS-containing proteoliposomes generated with a protein-to-lipid ratio of 1:1,000 was near monodisperse.

### CobS orientation in liposomes.

**FIG 8:**
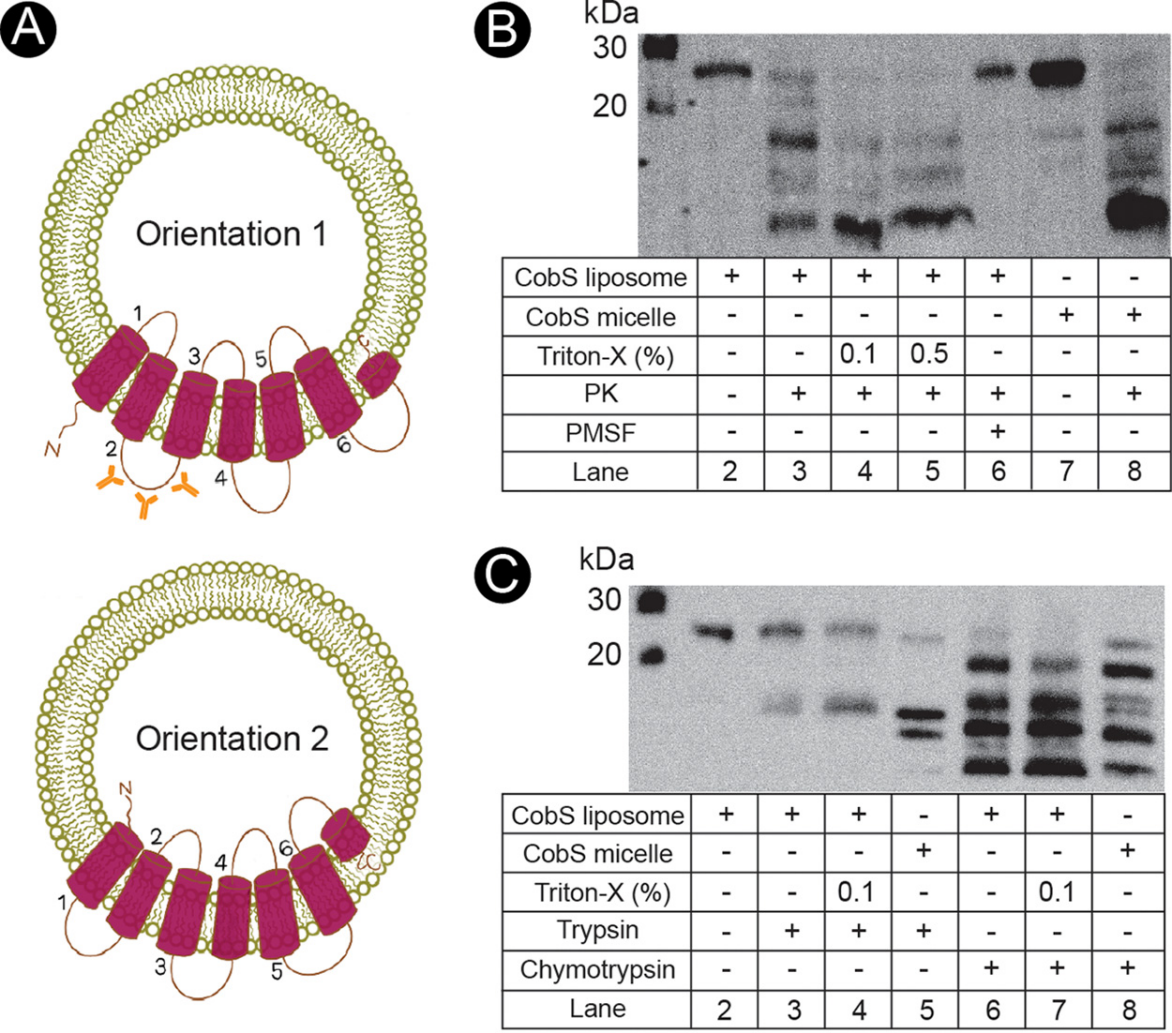
Determination of CobS orientation in liposomes by proteolysis. (A) Illustration of the two possible CobS orientations in the lipid bilayer of the liposome. Orientations 1 and 2 illustrate the possible orientations of CobS once inserted into liposomes. Anti-CobS antibodies used in subsequent proteolysis experiments are shown in orange along the region of CobS used to elicit the aforementioned polyclonal rabbit antibodies, i.e., cytoplasmic loop number two. (B) Western blot of proteolytic cleavage of CobS proteoliposome population by proteinase K (PK). Protease inhibitor PMSF was used as a control. (C) Western blot of proteolytic cleavage of CobS proteoliposome population by trypsin and chymotrypsin. Purified CobS was used as a control. Triton X-100 detergent was added at 0.1% (vol/vol) to disrupt the liposome and release CobS.

When generating proteoliposomes, we considered the two possible orientations in which CobS could incorporate in the lipid bilayer. First, the N terminus of CobS could extend from the outer surface of the proteoliposome or, alternatively, the N terminus of CobS could be found inside the lumen of the proteoliposome (Fig. 8A). To assess the orientation of the population of CobS-containing proteoliposomes, we performed proteolytic digests using proteinase K (PK).

Figure 8B shows results of Western blot experiments probing with polyclonal rabbit anti-CobS antibodies generated against loop 2; lane 1 served to control for molecular mass (kDa). CobS-containing proteoliposomes not treated with PK showed one band at ∼26 kDa (lane 2). Similarly, purified CobS (micelle) that was not treated with PK showed one major band at ∼26 kDa (lane 7). In addition to the major population of full-length CobS, we saw low levels of degraded CobS below 20 kDa (lane 7).

CobS-containing proteoliposomes and CobS digested with PK yielded multiple fragments below ∼26 kDa (lanes 3 and 8, respectively). CobS-containing proteoliposomes incubated with Triton X-100 (0.1% and 0.5% [vol/vol]) before PK digestion showed an increase in the intensity of low-molecular-mass fragments compared to PK-digested CobS-containing proteoliposomes not treated with Triton X-100 (lanes 4 and 5). We posited that incubation of proteoliposomes with detergent would disrupt the structure of the proteoliposome, making the cytoplasmic and periplasmic loops of the protein susceptible to digestion by PK. When the protease inhibitor phenylmethylsulfonyl fluoride (PMSF) was introduced, we saw complete inhibition of PK digestion of CobS-containing proteoliposomes (lane 6). The digestion pattern observed in the liposomes treated with PK was similar to the one observed with digested liposomes after detergent incubation, an observation that supported the conclusion that loop 2 was exposed, favoring the orientation where the N terminus was located outside the lumen of the proteoliposome.

In addition to digestion with PK, we digested loop 2 with trypsin and chymotrypsin (Fig. 8C). As before, lane 1 served to control for molecular mass (kDa). The undigested CobS-containing liposomes showed a single band at ∼26 kDa (lane 2). When digested with trypsin, we observed an additional fragment below 20 kDa alone with the full-length CobS at ∼26 kDa (lane 3). When incubated with Triton X-100, an increase in intensity below 20 kDa was also evident (lane 4). CobS digested with trypsin yielded three fragments below 20 kDa, suggesting that reconstitution in the lipid bilayer protected CobS from multiple trypsin cleavage sites (lane 5). Chymotrypsin digestion of CobS-containing proteoliposomes generated four major fragments: one ∼20 kDa and three smaller fragments (lane 6). When incubated with Triton X-100, we observed a decrease in intensity of the band at ∼20 kDa (lane 7). Digestion of purified CobS yielded additional fragments compared to CobS proteoliposomes, again suggesting occlusion (lane 8). Results of limited proteolysis were consistent with most of CobS being inserted into the liposome bilayer as shown in Fig. 8A, orientation 1.

### Affinity of CobS for its nucleotide substrate in the lipid bilayer.

**FIG 9:**
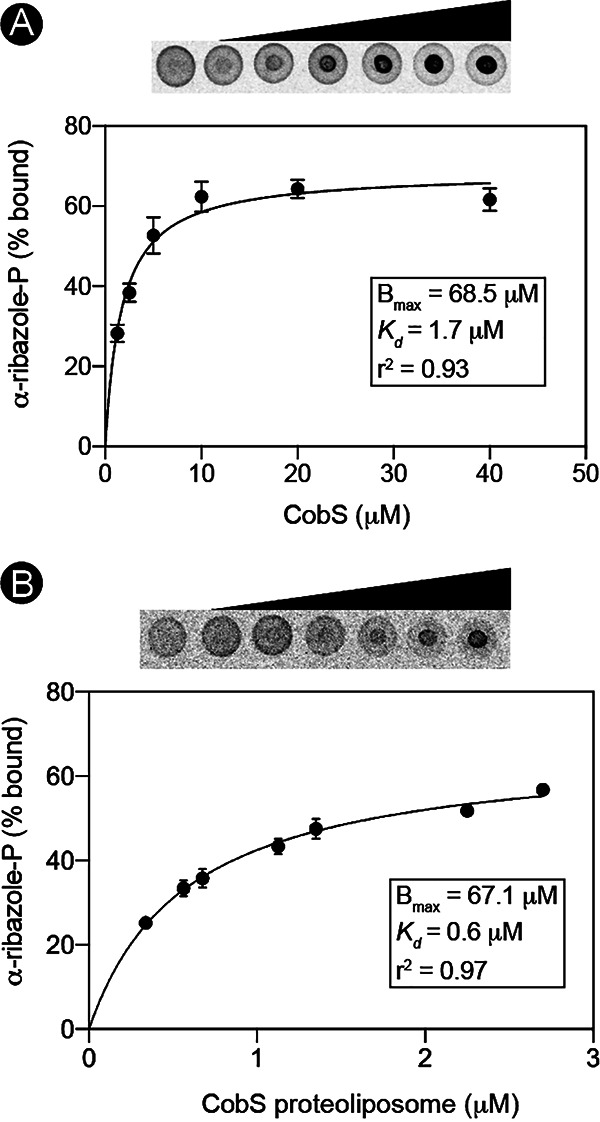
Determination of the *K_d_* of CobS for α-RP in proteoliposomes. (A) Increasing concentrations of purified CobS protein were incubated with [^14^C]α-RP (4.2 μM). Reaction mixtures were spotted onto a nitrocellulose membrane, and label distribution was visualized by phosphor imaging. Fraction of bound ligand was determined by differential radial capillary action of ligand assay (DRaCALA). Reactions were performed in biological and technical triplicates. Error bars represent standard errors of the means from technical triplicates. The dissociation constant (*K_d_*) was determined by nonlinear regression (GraphPad Prism v8). (B) Increasing concentrations of CobS-containing proteoliposomes were incubated with [^14^C]α-RP (420 nM). B_max_, maximum density of receptors.

In addition to determining the effect of a lipid environment on CobS activity, we investigated the effect of the same environment on substrate binding. Affinity for the α-RP substrate was determined by differential radial capillary action of ligand assay (DRaCALA) using a radiolabeled α-RP ligand (prepared as described in Text S1) incubated with 1,2-diheptanoyl-*sn*-glycero-3-phosphocholine (DHPC)-solubilized CobS or CobS-containing proteoliposomes (Fig. 9).

10.1128/mBio.00215-21.1 TEXT S1 Supplemental materials and methods. Download Text S1, PDF file, 0.1 MB. Copyright © 2021 Jeter and Escalante-Semerena. 2021 Jeter and Escalante-Semerena. https://creativecommons.org/licenses/by/4.0/ This content is distributed under the terms of the Creative Commons Attribution 4.0 International license.

Using this approach, we calculated that the dissociation constant (*K_d_*) of the CobS/α-RP complex was 1.71 μM, indicating a high affinity for the nucleotide substrate (Fig. 9A). The *K_d_* of the CobS-containing proteoliposome/α-RP complex was calculated to be 0.58 μM, a 3-fold decrease in *K_d_* compared to that for DHPC-solubilized CobS (Fig. 9B). The higher affinity of liposome-embedded CobS for the α-RP substrate was expected given the reported low intracellular concentration of the NaMN substrate of the CobT phosphoribosyltransferase that synthesizes α-RP (28). Due to the poor diffusion of AdoCbi-GDP (prepared as described in Text S1 and quantified using data shown in Fig. S2) on nitrocellulose, binding kinetics for this substrate were not obtained.

10.1128/mBio.00215-21.5 FIG S2 Adenosylcobinamide-GDP (AdoCbi-GDP) molar absorptivity. The figure shows the increase in the absorbance at 459 nm as a function of adenosylcobinamide-GDP (AdoCbi-GDP) concentration. Linear regression was performed using Prism v8 (GraphPad) to determine the molar absorptivity. Download FIG S2, PDF file, 0.4 MB. Copyright © 2021 Jeter and Escalante-Semerena. 2021 Jeter and Escalante-Semerena. https://creativecommons.org/licenses/by/4.0/ This content is distributed under the terms of the Creative Commons Attribution 4.0 International license.

### Nucleotide substrate binding induces a CobS conformational change.

**FIG 10:**
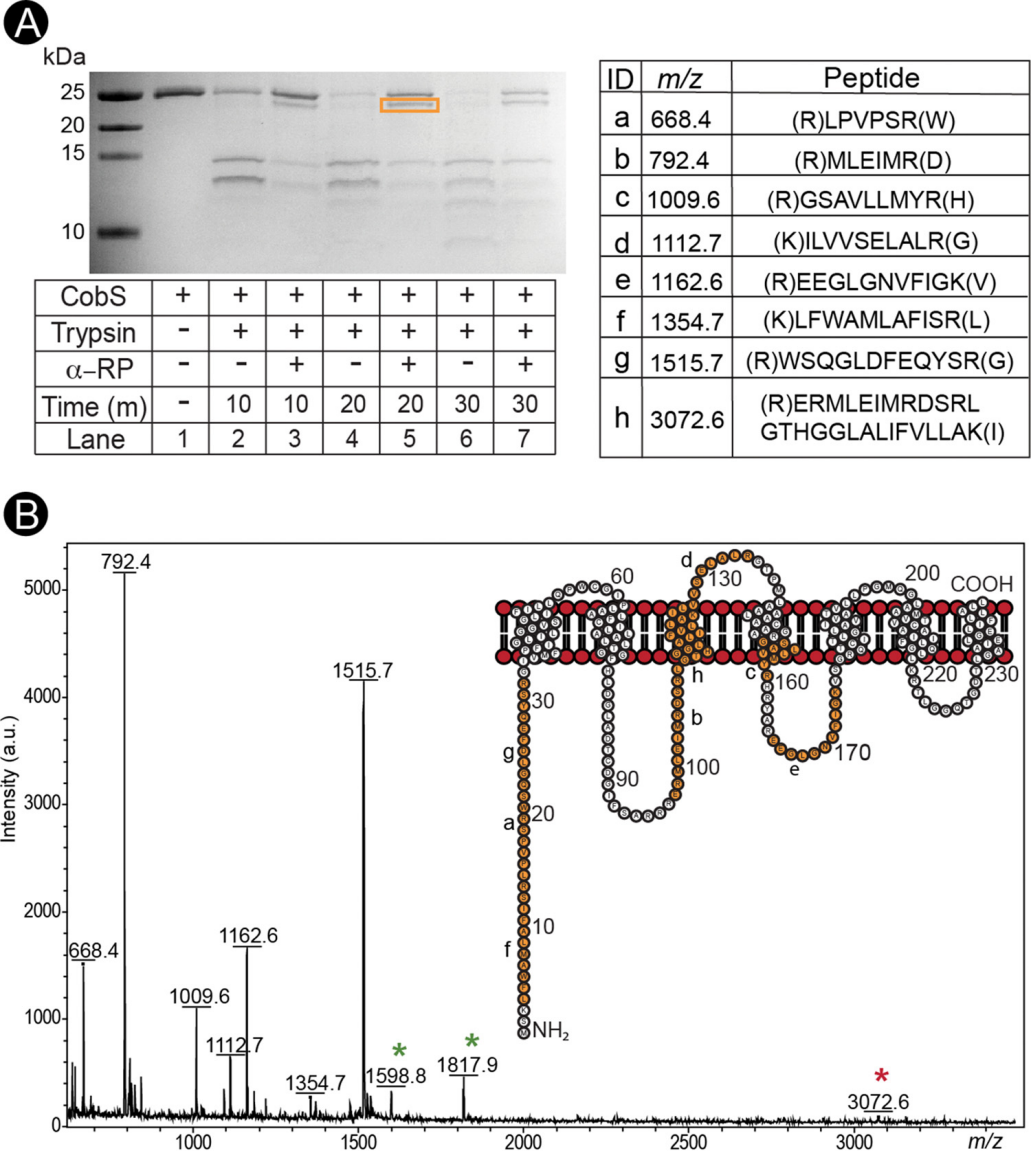
α-RP binding induces CobS conformational change. (A) Tricine-SDS-PAGE analysis of limited trypsin proteolysis of CobS and CobS preincubated with α-RP is shown. (B) MALDI-TOF analysis of the ∼24-kDa peptide generated from limited trypsin proteolysis on CobS preincubated with α-RP. Peaks are labeled with the corresponding mass and identified peptide fragment. Green asterisks indicated peaks that were not assigned. Red asterisk indicates a modification to the peptide sequence. Orange residues in the CobS topology inset correspond to fragments identified by MALDI-TOF analysis.

To assess the effect of substrate binding on CobS structure, CobS protein was subjected to limited trypsin proteolysis after incubation with α-RP and AdoCbi-GDP. Changes in protein structure were analyzed by tricine-SDS-PAGE (Fig. 10A) followed by matrix-assisted laser desorption ionization–time of flight (MALDI-TOF) mass spectrometry analysis (Fig. 10B). Preincubation with AdoCbi-GDP did not provide protection from proteolysis by trypsin. As shown in Fig. 10A, preincubation with α-RP resulted in the retention of full-length CobS protein and a prominent ∼24-kDa band over the course of a 30-min digestion (Fig. 10A, lane 5). CobS protein in the absence of α-RP was readily digested. MALDI-TOF mass spectrometry analysis of the ∼24-kDa peptide identified peptides from Leu^4^ to Lys^175^ (Fig. 10B). These data suggested that a significant CobS conformational change occurred upon α-RP binding.

### *In vivo* assessment of single-amino-acid variant CobS proteins.

**FIG 11:**
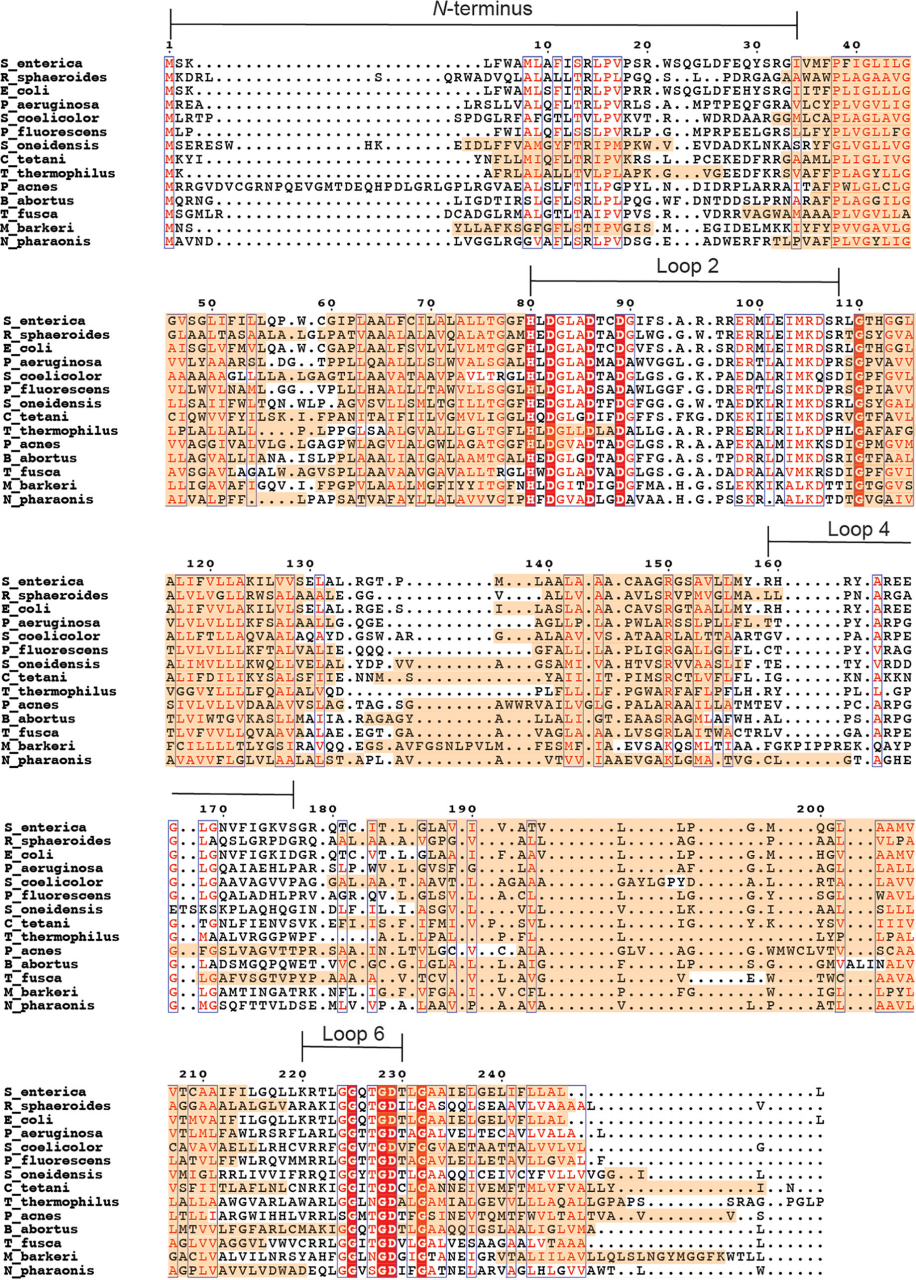
Conservation of membrane association among bacterial and archaeal cobamide synthases. The alignment was generated using T-COFFEE multiple sequence alignment server (PSI/TM-Coffee) and visualized with ESPript 3.0 web-based application. Transmembrane domains predicted by PSI/TM-COFFEE are highlighted with orange. Cytoplasmic loops *of* S. enterica CobS are labeled. Red highlight indicates amino acid residues conserved among all aligned organisms. Boxes indicate regions of high similarity.

All strains, plasmids, and primers used for this section of the work are reported in Tables S1 and S2. Given that the two enzymes that generate the substrates for CobS (i.e., CobU and CobT) (Fig. 2) are cytosolic proteins, we were interested in the conserved amino acids within cytoplasmic loops 2 and 6 (Fig. 3) of CobS proteins (Fig. 11, red highlights) as potential points of interaction with substrates, with CobU or CobT proteins, or for catalysis (for complete sequences of CobS proteins, see Fig. S3 in the supplemental material).

10.1128/mBio.00215-21.6 FIG S3 Conservation of membrane association among bacterial and archaeal cobamide synthases. The alignment was generated using T-COFFEE multiple sequence alignment server (PSI/TM-Coffee) and visualized with ESPript 3.0 web-based application. Transmembrane domains predicted by PSI/TM-COFFEE are highlighted with orange. Cytoplasmic loops of S. enterica CobS are labeled. Red highlight indicates amino acid residues conserved amongst all aligned organisms. Boxes indicate regions of high similarity. Download FIG S3, PDF file, 0.5 MB. Copyright © 2021 Jeter and Escalante-Semerena. 2021 Jeter and Escalante-Semerena. https://creativecommons.org/licenses/by/4.0/ This content is distributed under the terms of the Creative Commons Attribution 4.0 International license.

10.1128/mBio.00215-21.2 TABLE S1 Strains and plasmids used in this study. All *Salmonella* strains were derivatives of Salmonella enterica subsp. *enterica* serovar Typhimurium LT2. All strains used were constructed during the course of this work, unless otherwise indicated. Download Table S1, PDF file, 0.06 MB. Copyright © 2021 Jeter and Escalante-Semerena. 2021 Jeter and Escalante-Semerena. https://creativecommons.org/licenses/by/4.0/ This content is distributed under the terms of the Creative Commons Attribution 4.0 International license.

10.1128/mBio.00215-21.3 TABLE S2 Primers used in this work. All primers were purchased from Integrated DNA Technologies (IDT), Coralville, IA. Download Table S2, PDF file, 0.03 MB. Copyright © 2021 Jeter and Escalante-Semerena. 2021 Jeter and Escalante-Semerena. https://creativecommons.org/licenses/by/4.0/ This content is distributed under the terms of the Creative Commons Attribution 4.0 International license.

We queried the possible roles of some residues on substrate binding using site-directed mutagenesis of *cobS* cloned under the control of an l-(+)-arabinose inducible promoter. Using this approach, we generated alleles that encoded single-amino-acid CobS variants. Newly constructed vectors were transformed into *S.* Typhimurium strain JE8248 (*metE205 ara-9* Δ*cobS1313*) to assess CobS function *in vivo*. We assessed the growth of strains that synthesized CobS variants under Cba-dependent conditions (Table 1; see also Fig. S4 to S6). All strains tested carried a null allele of the Cba-independent methionine synthase (MetE) enzyme, hence demanding that the last step of methionine synthesis was performed by the Cba-dependent methionine synthase (MetH) enzyme. In addition, the strains carried a chromosomal deletion of *cobS*, making the strain ideal for the assessment of the function of CobS variants *in vivo*.

**TABLE 1:** Growth behavior of *S.* Typhimurium strains harboring plasmids encoding CobS variants^*a*^

Variant (plasmid)^*b*^	Growth rate (μ^*c*^) (lag time [h]) in:^*d*^						
	0.5 nM Cbi	0.5 nM Cbi + DMB	1 nM Cbi	1 nM Cbi + DMB	1.5 nM Cbi	1.5 nM Cbi + DMB	100 nm Cbl
WT	0.3 ± 0.02 (7)	0.6 ± 0.01 (6)	0.8 ± 0.03 (6)	0.7 ± 0.01 (6)	0.8 ± 0.01 (6)	0.7 ± 0.01 (6)	0.8 ± 0.02 (6)
Vector (**pBAD33**)	NG	NG	NG	NG	NG	NG	0.8 ± 0.01 (6)
Vector (pBAD24)	NG	NG	NG	NG	NG	NG	0.8 ± 0.03 (5)
CobS^WT^ (**pCOBS69**)	0.2 ± 0.03 (7)	0.6 ± 0.02 (6)	0.9 ± 0.05 (5)	0.7 ± 0.01 (5)	0.8 ± 0.01 (5)	0.7 ± 0.01 (5)	0.8 ± 0.03 (6)
CobS^WT^ (**pCOBS68**)	0.2 ± 0.01 (7)	0.5 ± 0.03 (7)	0.8 ± 0.01 (7)	0.7 ± 0.01 (7)	0.8 ± 0.02 (7)	0.7 ± 0.01 (7)	0.8 ± 0.01 (7)
CobS^G45E^ (**pCOBS82**)	NG	NG	NG	NG	NG	NG	0.8 ± 0.02 (6)
CobS^H80A^ (**pCOBS71**)	NG	0.6 ± 0.01 (6)	0.04 ± 0.01 (1)	0.7 ± 0.02 (6)	0.7 ± 0.2 (6)	0.8 ± 0.02 (7)	0.8 ± 0.02 (6)
CobS^D82A^ (**pCOBS89**)	NG	NG	NG	NG	NG	NG	0.8 ± 0.03 (6)
CobS^D86A^ (**pCOBS79**)	NG	NG	NG	NG	NG	NG	0.8 ± 0.03 (7)
CobS^D89A^ (**pCOBS78**)	NG	NG	NG	NG	NG	NG	0.8 ± 0.03 (7)
CobS^M104A^ (**pCOBS73**)	NG	0.6 ± 0.01 (6)	NG	0.7 ± 0.02 (7)	0.8 ± 0.02 (6)	0.8 ± 0.01 (7)	0.7 ± 0.02 (6)
CobS^R105K^ (pCOBS105)	0.2 ± 0.01 (5)	0.6 ± 0.01 (6)	0.6 ± 0.04 (7)	0.5 ± 0.01 (7)	0.6 ± 0.01 (6)	0.5 ± 0.03 (7)	0.7 ± 0.02 (6)
CobS^R108E^ (pCOBS109)	0.1 ± 0.01 (4)	0.7 ± 0.01 (6)	0.8 ± 0.04 (6)	0.8 ± 0.02 (6)	0.9 ± 0.01 (6)	0.8 ± 0.02 (6)	0.9 ± 0.03 (6)
CobS^R108K^ (pCOBS107)	0.1 ± 0.01 (4)	0.7 ± 0.01 (6)	0.7 ± 0.03 (6)	0.8 ± 0.03 (6)	0.9 ± 0.01 (6)	0.9 ± 0.02 (6)	0.9 ± 0.03 (6)
CobS^R159K^ (pCOBS108)	0.2 ± 0.02 (6)	0.6 ± 0.01 (6)	1.0 ± 0.02 (6)	0.9 ± 0.03 (6)	1.0 ± 0.02 (5)	0.8 ± 0.01 (6)	0.8 ± 0.01 (6)
CobS^R164K^ (pCOBS106)	0.1 ± 0.01 (4)	0.7 ± 0.01 (6)	0.4 ± 0.03 (6)	0.8 ± 0.04 (6)	0.9 ± 0.03 (6)	0.8 ± 0.02 (6)	0.8 ± 0.04 (6)
CobS^L168V^ (pCOBS104)	0.1 ± 0.01 (1)	0.6 ± 0.01 (6)	0.7 ± 0.03 (7)	0.6 ± 0.01 (6)	0.7 ± 0.01 (6)	0.6 ± 0.02 (6)	0.7 ± 0.01 (6)
CobS^D229A^ (**pCOBS83**)	NG	NG	NG	NG	NG	NG	0.8 ± 0.03 (7)
CobS^G225A^ (**pCOBS85**)	0.1 ± 0.01 (9)	0.6 ± 0.02 (6)	0.7 ± 0.01 (7)	0.9 ± 0.01 (7)	0.7 ± 0.01 (6)	0.7 ± 0.01 (6)	0.8 ± 0.03 (7)
CobS^G232A^ (**pCOBS84**)	NG	0.5 ± 0.01 (8)	0.5 ± 0.2 (8)	0.7 ± 0.01 (8)	0.7 ± 0.05 (8)	0.7 ± 0.01 (8)	0.8 ± 0.03 (7)

a All strains carried chromosomal *metE205* and *ara-9* mutations, which forced the cells to catalyze the last step of methionine synthesis (HCys-to-Met) via the cobamide-dependent methionine synthase (MetH) enzyme, and blocked arabinose catabolism, respectively. All strains were grown in no-carbon essential (NCE) minimal medium supplemented with glycerol (22 mM) as the sole carbon and energy source.

b CobS variants encoded by *cobS* alleles cloned into pBAD33 are in boldface font; CobS variants encoded by *cobS* alleles cloned into pBAD24 are shown in lightface font. Strain listed as WT is the parent strain without plasmid.

c Defined as ΔOD_630_ h^−1^.

d Growth rates are shown with the standard deviation from three replicates. The experiment was performed thrice. NG, no growth; Cbi, dicyanocobinamide [(CN)_2_Cbi]; DMB, 5,6-dimethylbenzimidazole; Cbl, cyanocobalamin (CNCbl, vitamin B_12_).

As shown in Fig. 3 and 11, within the first transmembrane domain (TM1) of CobS lies a conserved glycine residue at position 45. When a glutamate substitution was introduced at position 45, the resulting CobS^G45E^ variant failed to synthesize enough Cba 5′-phosphate product to support cell growth, despite the presence of Cbi and DMB precursors in the medium (Table 1; Fig. S4 to S6, solid green squares). This growth defect was overcome by the addition of cobalamin, indicating that G45 was critical for CobS function. Glycines, especially repeats as seen here, are often important for proper helix packing in the membrane. It is likely the glutamate substitution at this location interrupted the packing of TM1.

### (i) Cytoplasmic loop 2.

We noted that cytoplasmic loop 2 of all CobS homologues contained a motif flanked by conserved residues H80 and D89, with two more conserved aspartyl residues at positions 82 and 86 (^80^HxDxxxDxxD^89^) (Fig. 11). To probe the relevance of this motif to CobS function, we performed an alanine scan of the conserved residues within this motif. Under the conditions tested, alanine substitutions at residues D82, D86, and D89 resulted in abrogation of growth in medium supplemented with Cbi and DMB (Table 1) (D82A, Fig. S4 and S5, solid gray diamonds; D86A, Fig. S4 and S5, solid orange triangles; D89A Fig. S4 and S5, gray asterisks). In each of these strains, growth was restored when cobalamin was provided. Notably, the CobS^H80A^ variant did not synthesize enough AdoCbl-P to support growth of the tester strain when a low concentration of Cbi (0.5 nM) was added to the medium. However, the CobS^H80A^ variant supported growth when Cbi and DMB were present in the culture medium (Table 1; Fig. S4, open black squares). When the concentration of Cbi in the medium was increased to 1 nM, the strain that synthesized the CobS^H80A^ variant grew, albeit at a lower rate than wild-type CobS (CobS^WT^)-synthesizing strains (Table 1; Fig. S5A, open black squares). Increasing Cbi to 1.5 nM resulted in a growth rate comparable to that of a strain that synthesized CobS^WT^ (Table 1; Fig. S6A, open black squares). Restoration of growth of a strain synthesizing a CobS^H80A^ variant by increasing the concentration of ring precursor suggested that this loop may interact with the corrinoid ring substrate AdoCbi-GDP. Each of the conserved residues within this motif appear to significantly contribute to CobS function.

10.1128/mBio.00215-21.7 FIG S4 Growth behavior of *cobS* strains synthesizing variant CobS proteins in minimal medium supplemented with 0.5 nM Cbi. Strains were grown in minimal NCE medium supplemented with (CN)_2_Cbi (0.5 nM) and glycerol (22 mM) as a carbon and energy source. (B, D, and F) Strains were supplemented with DMB (150 μM). (A and B) CobS variants in TM1 and cytoplasmic loop 2. (C and D) CobS variants in cytoplasmic loop 4. (E and F) CobS variants in cytoplasmic loop 6. A strain synthesizing wild-type CobS in *trans* is depicted with open black circles. A *cobS*^+^ strain is depicted with closed red circles. Download FIG S4, PDF file, 0.5 MB. Copyright © 2021 Jeter and Escalante-Semerena. 2021 Jeter and Escalante-Semerena. https://creativecommons.org/licenses/by/4.0/ This content is distributed under the terms of the Creative Commons Attribution 4.0 International license.

10.1128/mBio.00215-21.8 FIG S5 Growth behavior of *cobS* strains synthesizing variant CobS proteins in minimal medium supplemented with 1 nM Cbi. Strains were grown in minimal NCE medium supplemented with (CN)_2_Cbi (1 nM) and glycerol (22 mM) as a carbon and energy source. (B, D, and F) Strains were supplemented with DMB (150 μM). (A and B) CobS variants in TM1 and cytoplasmic loop 2. (C and D) CobS variants in cytoplasmic loop 4. (E and F) include CobS variants in cytoplasmic loop 6. A strain synthesizing wild-type CobS in *trans* is depicted with open black circles. A *cobS*^+^ strain is depicted with closed, red circles. Download FIG S5, PDF file, 0.5 MB. Copyright © 2021 Jeter and Escalante-Semerena. 2021 Jeter and Escalante-Semerena. https://creativecommons.org/licenses/by/4.0/ This content is distributed under the terms of the Creative Commons Attribution 4.0 International license.

10.1128/mBio.00215-21.9 FIG S6 Growth behavior of *cobS* strains synthesizing different CobS variants in minimal medium supplemented with 1.5 nM Cbi. Strains were grown in minimal NCE medium supplemented with (CN)_2_Cbi (1.5 nM) and glycerol (22 mM) as a carbon and energy source. (B, D, and F) Strains were supplemented with DMB (150 μM). (A and B) CobS variants in TM1 and cytoplasmic loop 2. (C and D) CobS variants in cytoplasmic loop 4. (E and F) CobS variants in cytoplasmic loop 6. A strain synthesizing wild-type CobS in *trans* is depicted with open black circles. A *cobS*^+^ strain is depicted with closed red circles. Download FIG S6, PDF file, 0.5 MB. Copyright © 2021 Jeter and Escalante-Semerena. 2021 Jeter and Escalante-Semerena. https://creativecommons.org/licenses/by/4.0/ This content is distributed under the terms of the Creative Commons Attribution 4.0 International license.

At the end of loop 2, we noted an additional region of conservation between residues 98 and 106 (Fig. 11). When alanine was substituted for methionine at position 104, growth was observed in medium containing both Cbi and DMB but not low Cbi (0.5 nM and 1.0 nM) alone (Table 1; Fig. S4A and S5A, solid black circles). Increasing Cbi to 1.5 nM resulted in a growth rate comparable to that of a strain synthesizing CobS^WT^ (Table 1; Fig. S6A, solid black circles). When lysine was substituted for arginine at position 105, no significant changes in growth were observed (Table 1; Fig. S4A, S5A, and S6A, open orange diamonds). Interestingly, strains synthesizing both CobS^R108K^ and CobS^R108E^ exhibited a slight decrease in growth rate compared to that of strains synthesizing CobS^WT^ with low Cbi (0.5 nM) (Table 1) (R108K, Fig. S4A, open green triangles; R108E, Fig. S4A, open, inverted gray triangles). When DMB was provided, the slight defect in growth rate was no longer observed (Table 1) (R108K, Fig. S4B, open green triangles; R108E, Fig. S4B, open, inverted gray triangles). Given the marginal effect on growth observed when we substituted a negatively charged residue at position 108, we surmised that residue R108 was not directly involved in binding the nucleotide substrate. The cessation of growth observed when aspartyl residues in this motif were replaced by alanines suggested an inability to interact with the activated corrin ring substrate, AdoCbi-GDP. Collectively, these results suggested that the change to a hydrophobic side chain may hinder AdoCbi-GDP binding, and the presence of α-RP (an amphipathic molecule) helps correct the problem. In addition to these results, data obtained using DRaCALA and limited trypsin proteolysis suggested that α-RP binds first and that the conformational change CobS undergoes upon α-RP binding may increase the affinity of the enzyme for AdoCbi-GDP.

### (ii) Cytoplasmic loop 4.

We also investigated cytoplasmic loop 4, whose amino acid composition varies across CobS homologues. Strains that synthesized both CobS^L168V^ and CobS^R164K^ exhibited a decreased growth rate under low-Cbi conditions (0.5 nM) compared to that of a strain synthesizing CobS^WT^ (Table 1) (L168V, Fig. S4C, closed brown diamonds; R164K, Fig. S4C closed orange triangles). This growth defect was ameliorated upon the addition of DMB, suggesting that slight modifications of the residue side chain at these locations could be overcome by binding the nucleotide substrate α-RP (Table 1; Fig. S4D). Interestingly, under low-Cbi conditions (0.5 nM), a strain synthesizing CobS^R159K^ exhibited a higher growth rate than a strain synthesizing CobS^WT^ (Table 1; Fig. S4C, closed green squares). Residue R159 lies near the interface of the cytoplasm and inner membrane. It is possible that the change to lysine at this location increases affinity for the nucleotide substrate, conferring a growth advantage for strains synthesizing CobS^R159K^ relative to that of strains synthesizing CobS^WT^.

### (iii) Cytoplasmic loop 6.

Cytoplasmic loop 6 contains a region of highly conserved residues from 225 to 232, with a motif of ^225^GxxGDxxG^232^ observed among all cobamide producers (Fig. 11); noteworthy are two additional conserved residues within the motif, namely, G228 and D229. Strains that synthesized CobS^G225A^ exhibited growth on low Cbi (0.5 nM) but with a severe decrease in growth rate and increase in lag compared to those of strains synthesizing CobS^WT^ (Table 1; Fig. S4E, closed orange triangles). In the presence of DMB, growth was restored to a rate and lag comparable to those of a strain synthesizing CobS^WT^ (Table 1; Fig. S4F, closed orange triangles).

Strains that synthesized CobS^G232A^ failed to synthesize sufficient cobamide to support growth in medium supplemented with low Cbi (0.5 nM) only (Table 1; Fig. S4E, closed brown diamonds), but growth was restored in medium containing Cbi and DMB (Table 1; Fig. S4F, closed brown diamonds). A strain that synthesized CobS^D229A^ did not grow when Cbi and DMB were provided, but growth was restored with exogenous cobalamin (Table 1; Fig. S4E and F, S5E and F, and S6E and F, closed green squares). Given the observed phenotypes, the role of residue D229 appears to be more important to CobS function than those of other conserved residues within this motif.

### Concluding remarks.

Based on this work, CobS homologues in bacteria and archaea are likely localized to the cell membrane. At present, it is unclear what is the positive selection for the use of the cell membrane in the synthesis of cobamides, but the selection is strong enough to be maintained throughout evolution. It is remarkable that the alluded positive selection is applied on all cobamide producers, regardless of the environment they occupy. Consequences of this localization may be driven to optimize the synthesis and use of the cobamide; however, such cell localization may also have implications for interactions of cobamide producers with other members of the community they occupy.

We do not think it is a coincidence that the last step of the corrin ring biosynthetic branch of the pathway is also catalyzed by a polytopic integral membrane protein (i.e., CbiB). In our current working mode, the CbiB and CobS proteins anchor a multienzyme complex to the cell membrane for the purpose of increasing the efficiency of product formation, while reducing the risk of loss of intermediates to the cell milieu. At a minimum, the putative complex is likely composed of the five enzymes shown in Fig. 2, i.e., CbiB, CobU, CobT, CobC, and CobS. However, it is reasonable to consider that the complex may also include cobalt transporters, corrinoid transporters, and cobamide chaperones that deliver the coenzyme to the enzymes that use it. The complex may also contain a corrinoid adenosyltransferase, that would meet the need for adenosylated intermediates during *de novo* synthesis, but it would also ensure that exogenous corrinoids are readily adenosylated upon entry into the cell.

We are using the new knowledge reported here to probe for interactions among the putative enzymes of the complex. Ultimately, the goal is to reconstruct the complex and to visualize it using microscopy methodologies.

It is worth noting that recently, Ma and coworkers proposed that the cobamide synthase (CobS) enzyme of Vibrio cholerae may be a bifunctional enzyme that is also involved in nucleobase remodeling (29). Although this is an exciting possibility, *in vitro* data in support of this proposal are lacking. If this activity were validated, it would significantly expand the physiological role of cobamide synthase in some organisms.

## MATERIALS AND METHODS

### Protein overproduction and purification.

CobS overproduction and purification were based on methods described elsewhere (27) with modifications. CobS^WT^ protein was overproduced from plasmid pCOBS5 in strain JE6663 [E. coli C41(λDE3)] in 2-liter cultures of terrific broth (30). Protein synthesis was induced by the addition of isopropyl-β-d-thiogalactopyranoside (IPTG) at a final concentration of 500 μM in mid-log-phase cultures (optical density at 600 nm [OD_600_] of ∼0.6) growing at 37°C while shaking at 180 rpm in an Innova44 gyratory incubator (New Brunswick Scientific). After induction, cultures were grown for 3 h at 37°C while shaking at 180 rpm. Cultures were harvested by centrifugation at 4°C for 15 min at 6,000 × *g* in an Avanti J20-XPI refrigerated centrifuge equipped with a JLA-8.1000 rotor. Pelleted cells were stored at −20°C until used. For protein purification, cell pellets were thawed and resuspended in 30 ml of Tris-HCl buffer (100 mM, pH 7.9, at 24°C). Cells were lysed with a cell press in a cell disruptor (Constant Systems) set at 1.72 × 10^5^ kPa. A sample (0.5 ml) of a protease inhibitor cocktail (Sigma) was added to cell extracts (CFEs) to minimize CobS degradation during purification. CFEs were obtained after centrifugation at 4°C at 5,000 × *g* for 15 min. Cell membranes were obtained from a high-speed centrifugation at 75,000 × *g* for 90 min in an Avanti J-25I Beckman/Coulter refrigerated centrifuge equipped with a JA-25.50 rotor. Membranes were resuspended in 10 ml of Tris-HCl buffer (0.1 M, pH 7.9, at 24°C) with a glass homogenizer and were solubilized by the addition of 3-[(3-cholamidopropyl)-dimethylammonio]-1-propanesulfonate (CHAPS) detergent to a final concentration of 20 mM. The detergent-containing CFE was incubated on ice for 1 h and centrifuged at 4°C at 75,000 × *g* for 30 min to remove solubilized contaminants. The insoluble fraction containing CobS was resuspended in 10 ml of Tris-HCl buffer (0.1 M, pH 7.9, at 24°C) containing NaCl (0.5 M) and imidazole (20 mM) with a glass homogenizer and solubilized by the addition of 1,2-diheptanoyl-*sn*-glycero-3-phosphocholine (DHPC) to a final concentration of 15 mM. DHPC was added slowly to avoid denaturation of proteins. The CFE fraction containing detergent-solubilized CobS was incubated on ice for 1 h and centrifuged at 4°C at 75,000 × *g* for 30 min. A HisPur nickel-nitrilotriacetic acid (Ni-NTA) resin (2-ml bed volume; Thermo Scientific) was equilibrated with 10 bed volumes of water and 10 bed volumes of Tris-HCl buffer (100 mM, pH 7.9, at 24°C) containing NaCl (0.5 M) and imidazole (20 mM). CFE containing solubilized CobS protein was incubated with the HisPur resin at 4°C with nutation for a minimum of 2 h before pouring the slurry into a column (Kontes Flex-Column, 1.0 by 10 cm). The column was washed with five bed volumes of Tris-HCl buffer (0.1 M, pH 7.9, at 24°C) containing NaCl (0.5 M), imidazole (20 mM), and DHPC (15 mM) and then with five bed volumes of Tris-HCl buffer (0.1 M, pH 7.9, at 24°C) containing NaCl (0.5 M), imidazole (60 mM), and DHPC (15 mM). Proteins were eluted with five bed volumes of Tris-HCl buffer (0.1 M, pH 7.9, at 24°C) containing NaCl (0.5 M), imidazole (0.5 M), and DHPC (15 mM). Proteins in fractions of interest were resolved by sodium dodecyl sulfate-polyacrylamide gel electrophoresis (SDS-PAGE) (31), and CobS-containing fractions were applied to a 5-ml Zeba Spin desalting column (Thermo Fisher) to remove imidazole. Glycerol (10% [vol/vol], final concentration) was added to a desalted CobS sample, which was flash frozen in liquid N_2_ prior to storage at −80°C. The final buffer composition before storage was Tris-HCl buffer (0.1 M, pH 7.9, at 24°C) containing NaCl (150 mM) and DHPC (15 mM). Protein concentration was determined using a Bradford assay kit (Bio-Rad Laboratories). Protein homogeneity was determined by densitometry analysis of CobS SDS-PAGE behavior using TotalLab 100 software. A typical CobS preparation yielded 0.5 mg of protein/g of cells. CobS purified using this method is referred to as CobS micelle in the text, given the presence of DHPC.

### Liposome preparation and protein reconstitution.

The lipids used in the study were 1-palmitoyl-2-oleoyl-glycero-3-phosphocholine (POPC), 1-palmitoyl-2-oleoyl-*sn*-glycero-3-phosphoethanolamine (POPE), 1-palmitoyl-2-oleoyl-*sn*-glycero-3-phoshpo-l-serine (POPS), and Lissamine rhodamine B 1,2-dihexadecanoyl-*sn*-glycero-3-phosphoethanolamine (Rh-DHPE) (Fig. S1). Proteoliposomes were prepared using detergent-mediated reconstitution as described elsewhere (32). POPC–POPS–POPE–Rh-DHPE were combined at a molar ratio of 80:10:9.5:0.5 to yield 1 μmol lipid. Lipids were dried under a stream of N_2_ gas and spun in an Eppendorf VacFuge at 30°C for 1 h. The dried lipid film was hydrated in 500 μl of HEPES buffer (20 mM, pH 7.4) containing NaCl (0.5 M), CHAPS (40 mM), and glycerol (10% [vol/vol]) to a final lipid concentration of 2 mM. Detergent-solubilized CobS^WT^ was added at a lipid-to-protein molar ratio of 1,000:1 and incubated with rotation on a Benchmark RotoBot at 4°C for 1 h. Detergent removal and proteoliposome formation was achieved by four 20-h dialyses using Slide-A-Lyzer dialysis cassettes (20-kDa molecular mass cutoff) against HEPES buffer (20 mM, pH 7.4) containing NaCl (0.5 M) and glycerol (10% [vol/vol]). Dialysis was performed at a dialysate/sample volumetric ratio of 2,000:1. After dialysis, the proteoliposome suspension was applied to a HistoDenz (Sigma) gradient to perform a liposome flotation assay. The proteoliposome suspension was mixed with equal volumes HEPES buffer (20 mM, pH 7.4) containing NaCl (0.15 M), HistoDenz (80% [wt/vol]), and glycerol (10% [vol/vol]) and deposited in a Beckman Coulter ultracentrifuge tube (polyallomer, 13 by 51 mm). A 4-ml overlay of HEPES (20 mM, pH 7.4) containing NaCl (0.15 M), HistoDenz (30% [wt/vol]), and glycerol (10% [vol/vol]) was applied followed by a 200-μl overlay of HEPES buffer (20 mM, pH 7.4) containing NaCl (0.15 M) and glycerol (10% [vol/vol]). The gradient was subjected to centrifugation at 4°C for 3 h at 268,000 × *g* in a refrigerated Beckman Coulter Optima MAX-XP ultracentrifuge using an MLS-50 rotor. The size distribution of reconstituted liposomes was determined by dynamic light scattering in a 1.5-ml polystyrene cuvette using a Zetasizer Nano (Malvern Instruments). Lipid concentration of the reconstituted liposomes was determined by a standard curve generated from fluorescence of Rh-DHPE. Fluorescence measurements were read on a BioTek Gemini instrument set at an excitation wavelength of 540 nm and emission wavelength of 586 nm. Protein concentration in proteoliposomes was determined by generating a standard curve. Briefly, a range of known bovine γ-globulin (BGG) and lysozyme concentrations were separated by SDS-PAGE with unknown concentrations of CobS-containing proteoliposomes stained with Oriole fluorescent stain and analyzed using TotalLab TL100 software. The presence of CobS in liposomes was confirmed by Western blot and mass spectrometry analyses.

### Proteolytic digest of CobS-containing proteoliposomes.

To determine protein orientation in the lipid bilayer of the liposome, proteolytic digests were performed using proteinase K (Fisher), trypsin, and chymotrypsin (Sigma). Reaction mixtures in HEPES buffer (20 mM, pH 7.4) containing NaCl (0.15 M), glycerol (10% [vol/vol]), 5 μl CobS-containing proteoliposomes, and 1 μg protease were incubated for 1 h at 37°C. Cobamide synthase reactions were stopped by the addition of Tris-HCl buffer (63 mM, pH 6.8) containing SDS (2% [wt/vol]), glycerol (10% [vol/vol]), 2-mercaptoethanol (100 mM), and bromophenol blue (0.001% [wt/vol]), followed by incubation at 90°C for 5 min. Peptides were separated by SDS-PAGE and subsequently analyzed by Western blotting (33) with anti-CobS rabbit polyclonal antibodies (Envigo, IN). Purified CobS^WT^ protein was used as a positive control.

### Binding kinetics.

The binding kinetics of α-RP to CobS^WT^ was determined using the differential radial capillary action of ligand assay (DRaCALA) method described elsewhere (34). Briefly, 20-μl reaction mixtures containing various concentrations of CobS^WT^ protein or CobS^WT^-containing liposomes were incubated with radiolabeled α-RP (4.2 μM and 420 nM, respectively) in phosphate-buffered saline (PBS). Two microliters from each reaction mixture was spotted on a nitrocellulose membrane and allowed to dry for 2 h before exposing to a phosphor screen for 16 to 48 h. The distribution of radioactivity on the nitrocellulose membrane was visualized using an Amersham Typhoon 5 (GE Life Sciences) with ImageQuant TL 8.1. The pixel density was analyzed using one-dimensional array analysis in TotalLab TL100 (Nonlinear Dynamics), and the resulting data were analyzed using Prism version 8 (GraphPad). Each reaction was performed in triplicates. The dissociation constant (*K_d_*) was determined using one-site-binding (hyperbola) nonlinear regression in Prism version 8 (GraphPad).

### *In vitro* cobamide synthase assay.

A continuous *in vitro* activity assay of cobamide synthase activity was implemented by modifying the conditions described elsewhere (27). To determine the specific activity of purified CobS protein and CobS-containing liposomes, the decrease in absorbance of AdoCbi-GDP was monitored at 459 nm for 5 min in a 96-well quartz microtiter plate using a SpectraMax Plus UV-visible spectrophotometer (Molecular Devices). CobS activity was calculated using the calculated AdoCbi-GDP molar absorptivity (ε_459_ = 3,352 M^−1 ^cm^−1^) using Beer-Lambert’s law equation, A = εcl. Data presented here represent technical triplicates of one experiment, which was repeated thrice.

Additional details on the methods utilized here are reported in the supplemental material (Text S1).

### Data availability.

All the data generated in this study are included in the paper.
