# Supplementary figures and images for: Insights into the Relationship between Cobamide Synthase and the Cell Membrane
Source: mBio. 2021 Mar 23;12(2):e00215-21. doi: 10.1128/mBio.00215-21 (PMC8092220; doi:10.1128/mBio.00215-21)

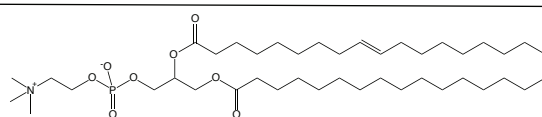

POPC

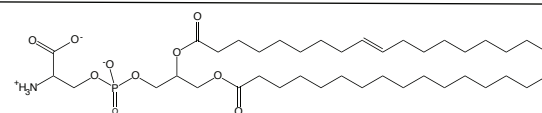

POPS

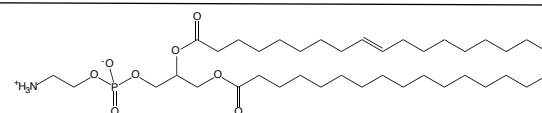

POPE

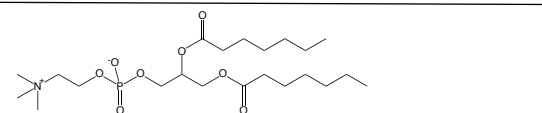

DHPC

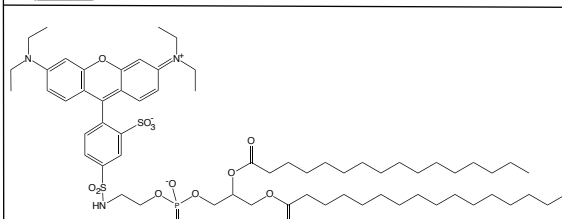

Rh-DHPE

Supplement: FIG S1 [file mBio.00215-21-sf001.pdf]

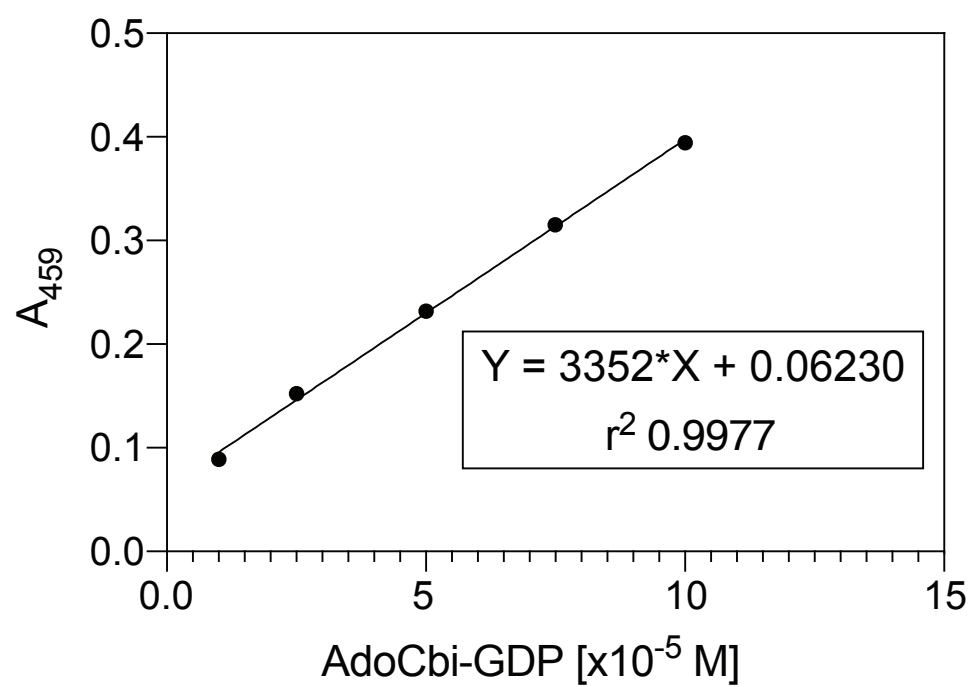

Supplement: FIG S2 [file mBio.00215-21-sf002.pdf]

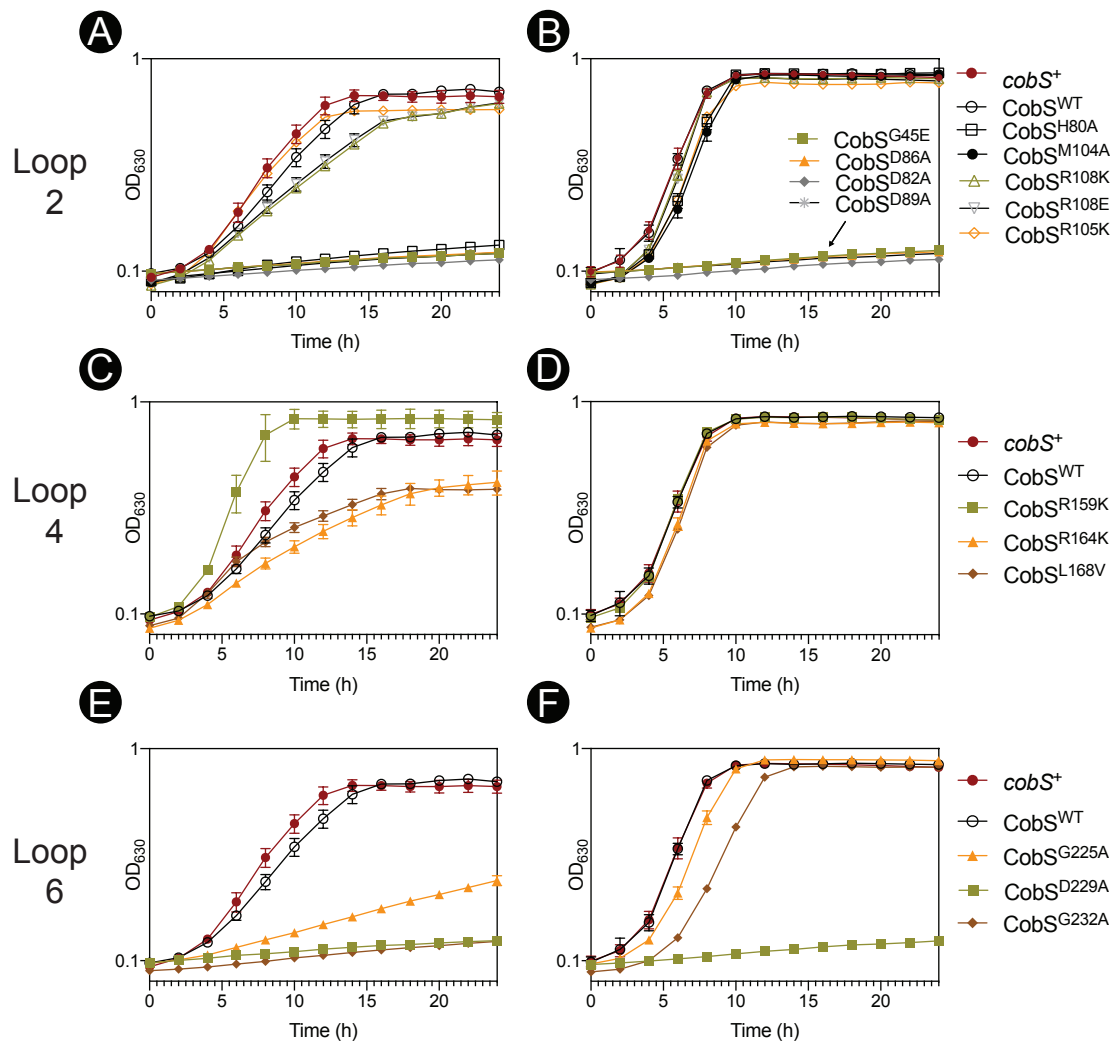

Supplement: FIG S4 [file mBio.00215-21-sf004.pdf]

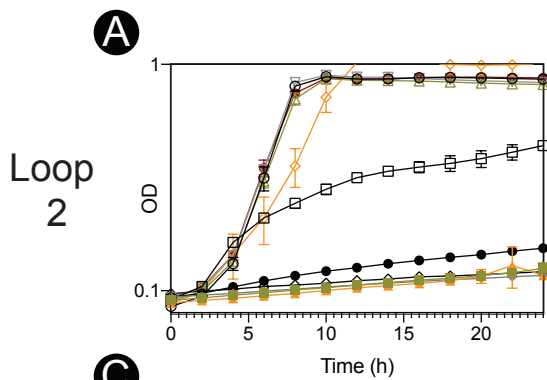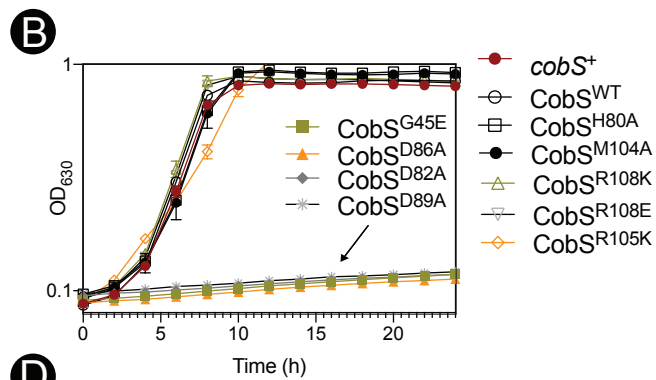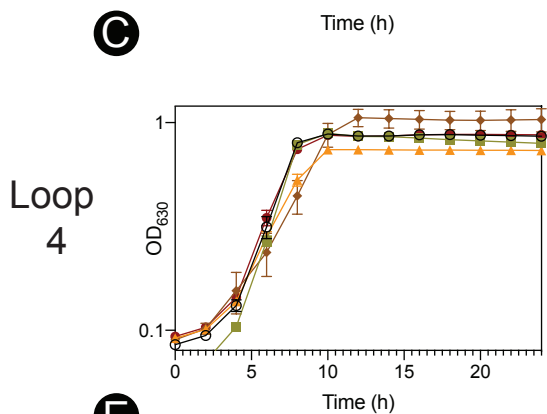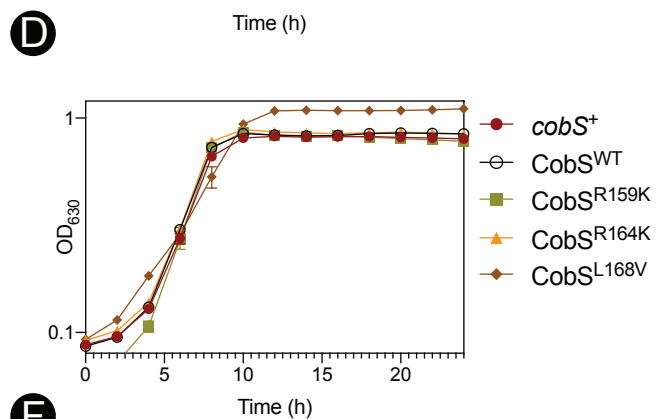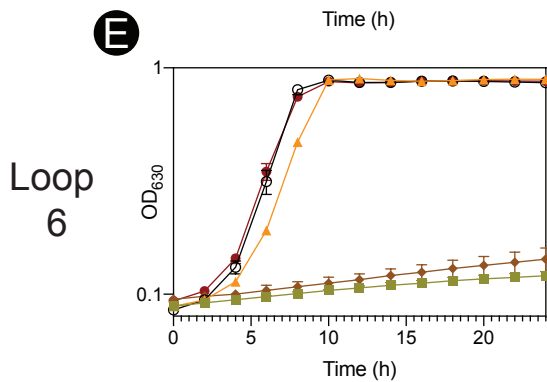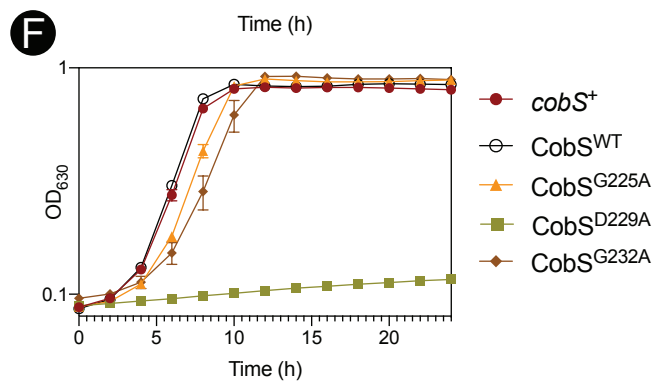

Supplement: FIG S5 [file mBio.00215-21-sf005.pdf]

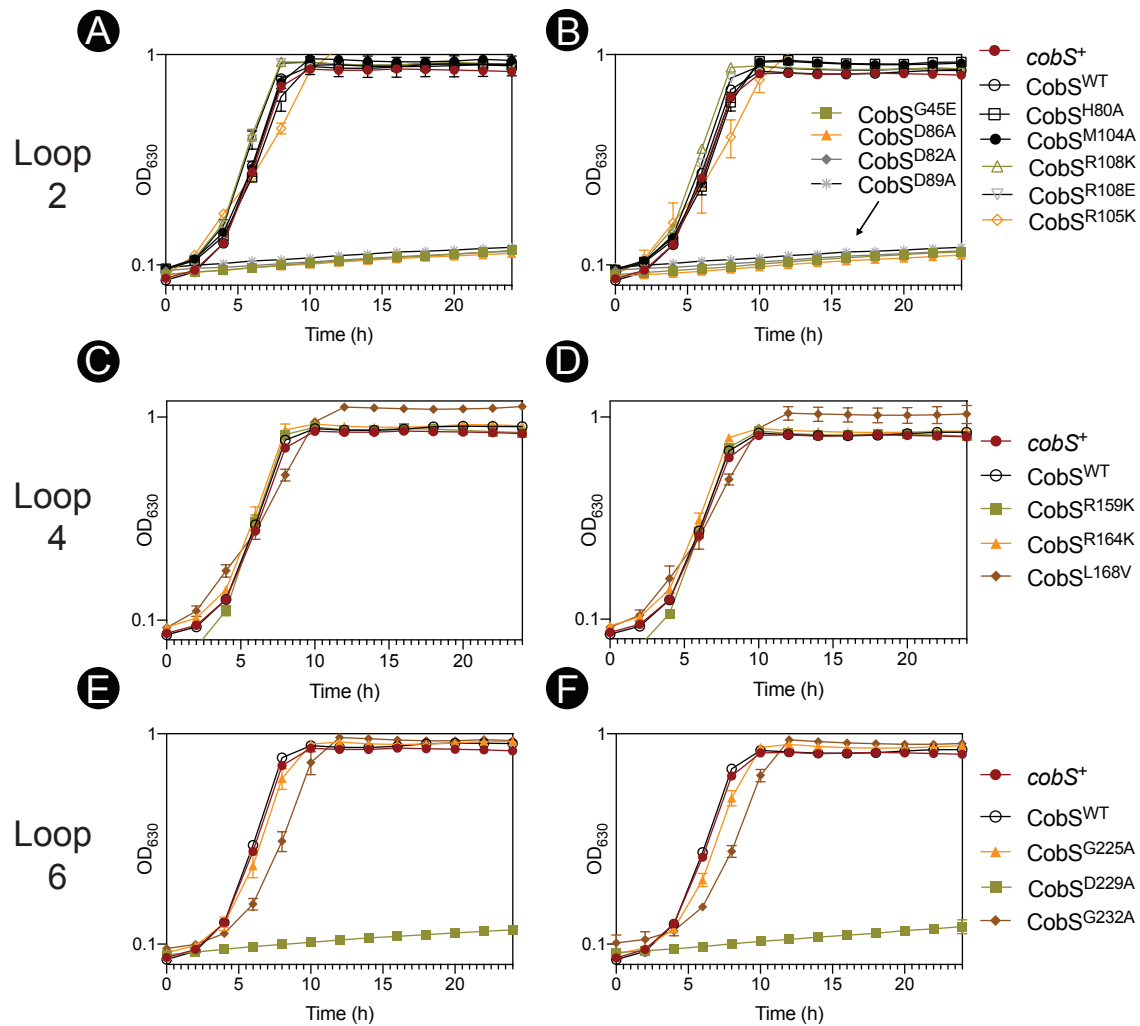

Supplement: FIG S6 [file mBio.00215-21-sf006.pdf]
