# Supplementary material for: Insights into the Relationship between Cobamide Synthase and the Cell Membrane
Source: mBio. 2021 Mar 23;12(2):e00215-21. doi: 10.1128/mBio.00215-21 (PMC8092220; doi:10.1128/mBio.00215-21)
Supplement: TEXT S1 [file mBio.00215-21-s0001.pdf]

SUPPLEMENTAL MATERIAL

Insights into the relationship between cobamide synthase and the cell membrane

Victoria L. Jeter and Jorge C. Escalante-Semerena*

*Corresponding author: Department of Microbiology, University of Georgia, 212C Biological Sciences Building, 120 Cedar Street, Athens, GA 30602, USA, T: 1 (706)-542-2651, F: 1 (706)-
542-2815; Email: jcescala@uga.edu, URL: www.escalab.com

Running title: Cell membrane-dependent coenzyme B_{12} biosynthesis

MATERIALS AND METHODS

Bacterial strains, culture media, and chemicals. All chemicals were purchased from Sigma-Aldrich unless otherwise noted. Bacterial strains used in this study are listed in Table 1. All strains for growth analysis were derivatives of *Salmonella enterica enterica* sv Typhimurium LT2 (*S.* Typhimurium) or *Escherichia coli* C41 (λDE3). *S.* Typhimurium strains were grown at 37 °C on lysogeny broth (LB, Difco) (1, 2) or no-carbon essential (NCE) minimal medium (3) supplemented with glycerol (22 mM), $MgSO_4$ (1 mM), and Wolfe's trace minerals (4). When added to the medium, the precursor Cbi (added as $(CN)_2$Cbi), was at a concentration of 0.5, 1, and 1.5 nM, the DMB nucleobase was at a concentration of 150 μM, and Cbl (added as CNCbl) was at a concentration of 100 nM. *Escherichia coli* C41 (λDE3) (5) was used for protein overexpression and *E. coli* K12 strain DH5α (New England Biolabs) was used for plasmid construction. All *E. coli* strains were grown at 37°C in LB medium. Antibiotics for all media were used at the following concentrations: ampicillin, 100 μg mL^{-1}; chloramphenicol, 20 μg mL^{-1}; isopropyl β-D-1-thiogalactopyranpside (IPTG, Gold BioTechnology), glycerol (Fisher), 4-(2-hydroxyethyl)-1-piperazineethanesulfonic acid buffer (HEPES, Gold BioTechnology) 3-[(3-Cholamidopropyl)dimethylammonio]-1-propanesulfonate detergent (CHAPS, Gold BioTechnology), 1-palmitoyl-2-oleoyl-glycero-3-phosphocholine (POPC, Avanti Polar Lipids), 1-palmitoyl-2-oleoyl-*sn*-glycero-3-phoshpo-L-serine (POPS, Avanti Polar Lipids), 1-palmitoyl-2-oleoyl-*sn*-glycero-3-phosphoethanolamine (POPE, Avanti Polar Lipids), Lissamine™ Rhodamine B 1,2-Dihexadecanoyl-*sn*-glycero-3-phosphoethanolamine (Rh-DHPE, Molecular Probes) (for chemical structures see Fig. S1), Oriole Fluorescent Gel Stain (Bio-Rad Laboratories).

Plasmid construction. Plasmids used in this study are listed in Table S1. Primers were synthesized by Integrated DNA Technologies, Inc. (IDT [Coralville, IA, United States]) and are listed in Table S2. Genes were amplified from *S. enterica* genomic DNA using Phusion DNA polymerase (ThermoFisher) as per manufacturer's instructions. Plasmids encoding CobS variants were amplified using PfuUltra II DNA (Agilent Technologies) polymerase as per manufacturer's instructions. Restriction enzymes were purchased from Fermentas.

General strategy used to construct plasmids encoding CobS variants of interest. Plasmid encoding CobS variants were constructed using primers shown in Table S2 and plasmid pCOBS68 DNA as the template. Briefly, the primers encoding a specific substitution change were used to amplify *cobS* encoding plasmids pCOBS68 or pCOBS69. Amplification reactions were incubated with DpnI for 20 h at 37°C to digest the nonmutated parental DNA template.

The amplification product was then transformed in *E. coli* DH5α cells, and its nucleotide sequence was verified by Sanger sequencing. Using this strategy, we generated *cobS* alleles encoding variants $CobS^{G45E}$, $CobS^{H80A}$, $CobS^{D82A}$, $CobS^{D86A}$, $CobS^{D89A}$, $CobS^{M104A}$, $CobS^{G225A}$, $CobS^{D229A}$, $CobS^{G232A}$ from pCOBS68 and variants $CobS^{L168V}$, $CobS^{R105K}$, $CobS^{R108K}$, $CobS^{R108E}$, $CobSR^{159K}$, $CobS^{R164K}$ from pCOBS69. Plasmids encoding these variants are described in Table S1.

Strain construction and growth analysis. In-frame deletions of *S. enterica* genes were constructed using the phage lambda Red recombinase system as described (6). Plasmids were transformed into strains for complementation studies as described elsewhere (7). For growth analyses, 2-mL starter cultures in 13 x 100 mm borosilicate cultures tubes were grown 16 h at 37 °C with shaking at 180 rpm in LB containing appropriate antibiotic. Growth experiments were performed in 96-well microtiter dishes (Falcon), with each well containing 198 μl of medium plus 2 μl of inoculum. In some cases, the inoculum size was varied. The growth behavior of three technical replicates of each strain was analyzed, and the experiment was performed thrice. Table 1 reports representative growth rates with standard deviations of technical triplicates. Growth was monitored using a computer-controlled plate reader (BioTek, Model Eon). The optical density at 630 nm was measured every 30 min for a total time of 24 h; the microtiter dish was shaken between measurements. Data were analyzed using GraphPad Prism version 8. Growth rate and lag were calculated using the Gompertz curve fitting model [Source code (https://github.com/scott-saunders/growth_curve_fitting/blob/master/growth_curve_fitting_ver0.2.Rmd)]. Figure legends indicate whether or not the inoculum used was greater than 1% (v/v). L-(+)-Arabinose was used as inducer when indicated.

Mass spectrometry. Confirmation of protein identity was performed by the Proteomics and Mass Spectrometry (PAMS) core facility at the University of Georgia. Briefly, protein or liposome preparations were separated using SDS-PAGE. Bands of interest were excised and subjected to digest by trypsin and analyzed by MALDI-TOF mass spectrometry. Mass spectrometry results were compared to information in the MASCOT database (http://www.matrixscience.com/) to determine protein identity.

Western blot analysis. To confirm the presence of CobS in liposomes and to analyze proteolytic digests, western blots were performed using rabbit polyclonal antibodies generated against a synthetic peptide of cytoplasmic loop 2 (hereafter α-CobS) (8). Liposomes harvested from the floatation assay previously described or proteolytic digests were resolved by SDS-PAGE and subsequently transferred to a polyvinylidene fluoride (PVDF) membrane (Millipore). The transfer was performed using a Trans-Blot Turbo system (Bio-Rad Laboratories) set to StandardSD mini gel (1.0 A, 25 V, 30 min) using Tris-HCl transfer buffer (25 mM, pH 8) containing glycine (192 mM) and methanol (10% v/v)]. Membranes were incubated for 30 min in blocking buffer of phosphate buffered saline containing Tween 20 (PBST) comprised of NaH_2PO_4 (10 mM, pH 7.2), NaCl (0.9% w/v), Tween 20 (0.1% v/v), and instant dry milk (5%, w/v). Membranes were probed with α-CobS antibodies (1:5,000 in blocking buffer) for 1 h, then washed thrice (30 min each) with PBST. Membranes were then probed for 1 h with horseradish peroxidase (HRP)-conjugated goat α-rabbit secondary antibodies (Sigma) in PBST (1:10,000) before three, 30-min washes with PBST. Membranes were incubated in SuperSignal West Pico PLUS chemiluminescent substrate (ThermoFisher) for 2 min and imaged using a UVP

ChemStudio imaging instrument (AnalytikJena). Purified $CobS^{WT}$ protein was used as positive control and SuperSignal Molecular Weight Protein Ladder (ThermoFisher) was used as reference for the electrophoretic behavior of molecules of known molecular masses.

Limited trypsin proteolysis. An α-RP- induced conformational change of CobS was determined by limited trypsin proteolysis. The assay was performed based on the protocol outlined elsewhere (9) with modifications. Twenty-μL reactions were performed in Tris-HCl buffer (0.1 M, pH 7.9 @ 24°C) containing NaCl (0.15 M) and DHPC (15 mM) using diphenylcarbamyl chloride-treated trypsin. When provided, substrates were at a final concentration of 50 μM and pre-incubated for 10 min prior to digestion by trypsin. Protein composition of the reaction mixture was analyzed by tricine-SDS PAGE using a 16% gel (10). Peptide fragments were excised from the gel and in-gel tryptic digest followed by MALDI-TOF mass spectrometry analysis was performed by the PAMS facility at the University of Georgia. The MASCOT database was used to analyze peptide fingerprint data.

AdoCbi-GDP and α-RP substrate preparation. The two substrates of CobS, namely AdoCbi-GDP and α-RP were not commercially available and were synthesized as described elsewhere (11, 12). Briefly, dicyanocobinamide [$(CN)_2$Cbi] was incubated with ATP and homogeneous ATP:Co(I)rrinoid adenosyltransferase CobA protein purified as described (13). The reaction mixture was incubated for 16 h at 37 °C under dark, anoxic conditions. The product of the reaction, adenosylcobinamide (AdoCbi) was separated from other reaction components using a Waters Sep-Pak C18 column. AdoCbi was eluted off the column with 100% methanol. Methanol was removed using an Eppendorf VacFuge for 16 h. Dried AdoCbi was resuspended in a reaction mixture containing GTP and homogeneous NTP:$(HO)_2$Cbi kinase, GTP:AdoCbi guanylyltransferase CobU enzyme purified as described elsewhere (14) and incubated at 37 °C for 16 h in the dark to yield AdoCbi-GDP. AdoCbi-GDP was separated from reaction components using a Waters Sep-Pak C18 column, and AdoCbi was eluted off the column with 100% methanol. Methanol was removed using an Eppendorf VacFuge for 16 h. AdoCbi-GDP was resuspended in water and its identity confirmed by its UV-visible spectrum. AdoCbi-GDP was quantified by reading the absorbance at 367 nm after incubation with KCN at 90 °C for 10 min.

α–RP was synthesized as described elsewhere (7). Briefly, DMB was incubated with NaMN and homogeneous NaMN:DMB phosphoribosyltransferase CobT enzyme (15) 16 h at 37°C. α–RP was separated from reaction components by HPLC as described (16). Fractions containing α–RP were pooled, applied to a Waters Sep-Pak C18 column and eluted with 100 % methanol. Methanol was removed by 16h vacuum centrifugation using an Eppendorf VacFuge, and α–RP was quantified by reading absorbance at 280 nm.

Determination of the molar absorptivity of AdoCbi-GDP. The molar absorptivity of AdoCbi-GDP was determined by monitoring absorbance at 459 nm across a range of concentrations. A linear regression was generated from absorbance as a function of AdoCbi-GDP concentration using GraphPad Prism v8 software (Fig. S2). The molar absorptivity at 459 nm was determined to be 3352 M^{-1} cm^{-1}.

HPLC analysis of reaction products. KCN was added to reaction mixtures to a final concentration of 2.4 μM, and the samples were incubated for 10 min at 90 ºC. Cyanated

corrinoids were applied to a SpinX column (Costar, 0.45 μM cellulose acetate), eluted with methanol and diluted 10-fold in a 1:4 ratio of buffer B [KH_2PO_4 (0.1 M, pH 8) containing KCN (10 mM) and acetonitrile (50%, v/v)] to buffer C [KH_2PO_4 (0.1 M, pH 6.5) containing KCN (10 mM) in preparation for separation by HPLC. Cobamide 5'-P synthase reaction products were resolved by RP-HPLC using a Shimadzu Prominence UFLC SPD-M30A instrument equipped with a Phenomenex Synergi 4μ hydroRP80A 150 mm x 4.6 mm LC column as described (17, 18) with modifications as outlined elsewhere (19). Authentic CNCbl 5'-P was used HPLC as positive control. CNCbas were detected at 367 and 525 nm and eluted at 19 minutes.

Bioassay. A 2-mL starter culture of strain JE8248 (Δ*cobS*) in 13 x 100 mm borosilicate cultures tubes was grown 16 h at 37 °C with shaking at 180 rpm in LB. The starter culture (0.1 ml) was added to 4 ml of soft agar agar (0.5% w/v), gently mixed, and poured on a no-carbon essential (NCE) minimal medium (3) agar (1% w/v) plate supplemented with glycerol (22 mM), $MgSO_4$ (1 mM), and Wolfe's trace minerals (4). Cobamide synthase reactions containing CobS proteoliposomes were performed as outlined in the Materials and Methods in the main text. A 2 μL spot of cobamide synthase reaction passed through a 0.22-μm filter was applied to the plate. A 2-μL aliquot of cyanocobalamin (100 nM) was used as the positive control. A cobamide synthase reaction without enzyme was used as the negative control.

REFERENCES

1. Bertani G. 1951. Studies on lysogenesis. I. The mode of phage liberation by lysogenic *Escherichia coli*. J Bacteriol 62:293-300.
2. Bertani G. 2004. Lysogeny at mid-twentieth century: P1, P2, and other experimental systems. J Bacteriol 186:595-600.
3. Berkowitz D, Hushon JM, Whitfield HJ, Jr., Roth J, Ames BN. 1968. Procedure for identifying nonsense mutations. J Bacteriol 96:215-220.
4. Balch WE, Wolfe RS. 1976. New approach to the cultivation of methanogenic bacteria: 2-mercaptoethanesulfonic acid (HS-CoM)-dependent growth of *Methanobacterium ruminantium* in a pressurized atmosphere. Appl Environ Microbiol 32:781-791.
5. Miroux B, Walker JE. 1996. Over-production of proteins in *Escherichia coli*: mutant hosts that allow synthesis of some membrane proteins and globular proteins at high levels. J Mol Biol 260:289-298.
6. Datsenko KA, Wanner BL. 2000. One-step inactivation of chromosomal genes in *Escherichia coli* K-12 using PCR products. Proc Natl Acad Sci USA 97:6640-6645.
7. Jeter VL, Mattes TA, Beattie NR, Escalante-Semerena JC. 2019. A new class of phosphoribosyltransferases involved in cobamide biosynthesis is found in methanogenic archaea and cyanobacteria. Biochemistry 58:951-964.
8. Maggio-Hall LA, Claas KR, Escalante-Semerena JC. 2004. The last step in coenzyme B(12) synthesis is localized to the cell membrane in bacteria and archaea. Microbiology 150:1385-1395.
9. Gupta S, Chakraborti PK, Sarkar D. 2005. Nucleotide-induced conformational change in the catalytic subunit of the phosphate-specific transporter from *M. tuberculosis*: implications for the ATPase structure. Biochim Biophys Acta 1750:112-121.
10. Schagger H. 2006. Tricine-SDS-PAGE. Nat Protoc 1:16-22.

11. Maggio-Hall LA, Escalante-Semerena JC. 1999. In vitro synthesis of the nucleotide loop of cobalamin by *Salmonella typhimurium* enzymes. Proc Natl Acad Sci U S A 96:11798-11803.
12. Zayas CL, Escalante-Semerena JC. 2007. Reassessment of the late steps of coenzyme B_{12} synthesis in *Salmonella enterica*: Evidence that dephosphorylation of adenosylcobalamin-5'-phosphate by the CobC phosphatase is the last step of the pathway. J Bacteriol 189:2210-2218.
13. Suh S, Escalante-Semerena JC. 1995. Purification and initial characterization of the ATP:corrinoid adenosyltransferase encoded by the *cobA* gene of *Salmonella typhimurium*. J Bacteriol 177:921-925.
14. Thomas MG, Thompson TB, Rayment I, Escalante-Semerena JC. 2000. Analysis of the adenosylcobinamide kinase/adenosylcobinamide-phosphate guanylyltransferase (CobU) enzyme of *Salmonella typhimurium* LT2. Identification of residue His-46 as the site of guanylylation. J Biol Chem 275:27576-27586.
15. Trzebiatowski JR, Escalante-Semerena JC. 1997. Purification and characterization of CobT, the nicotinate-mononucleotide:5,6-dimethylbenzimidazole phosphoribosyltransferase enzyme from *Salmonella typhimurium* LT2. J Biol Chem 272:17662-17667.
16. Mattes TA, Escalante-Semerena JC. 2018. Facile isolation of alpha-ribazole from vitamin B_{12} hydrolysates using boronate affinity chromatography. J Chromatogr B Analyt Technol Biomed Life Sci 1090:52-55.
17. Blanche F, Thibaut D, Couder M, Muller JC. 1990. Identification and quantitation of corrinoid precursors of cobalamin from *Pseudomonas denitrificans* by high-performance liquid chromatography. Anal Biochem 189:24-29.
18. Chan CH, Escalante-Semerena JC. 2011. ArsAB, a novel enzyme from *Sporomusa ovata* activates phenolic bases for adenosylcobamide biosynthesis. Mol Microbiol 81:952-967.
19. Mattes TA, Escalante-Semerena JC. 2017. *Salmonella enterica* synthesizes 5,6-dimethylbenzimidazolyl-(DMB)-alpha-riboside. Why some Firmicutes do not require the canonical DMB activation system to synthesize adenosylcobalamin. Mol Microbiol 103:269-281.
20. Rocco CJ, Dennison KL, Klenchin VA, Rayment I, Escalante-Semerena JC. 2008. Construction and use of new cloning vectors for the rapid isolation of recombinant proteins from *Escherichia coli*. Plasmid 59:231-237.
21. Guzman LM, Belin D, Carson MJ, Beckwith J. 1995. Tight regulation, modulation, and high-level expression by vectors containing the arabinose PBAD promoter. J Bacteriol 177:4121-30.
