# Supplementary material for: Insights into the Relationship between Cobamide Synthase and the Cell Membrane
Source: mBio. 2021 Mar 23;12(2):e00215-21. doi: 10.1128/mBio.00215-21 (PMC8092220; doi:10.1128/mBio.00215-21)
Supplement: FIG S3 [file mBio.00215-21-sf003.pdf]

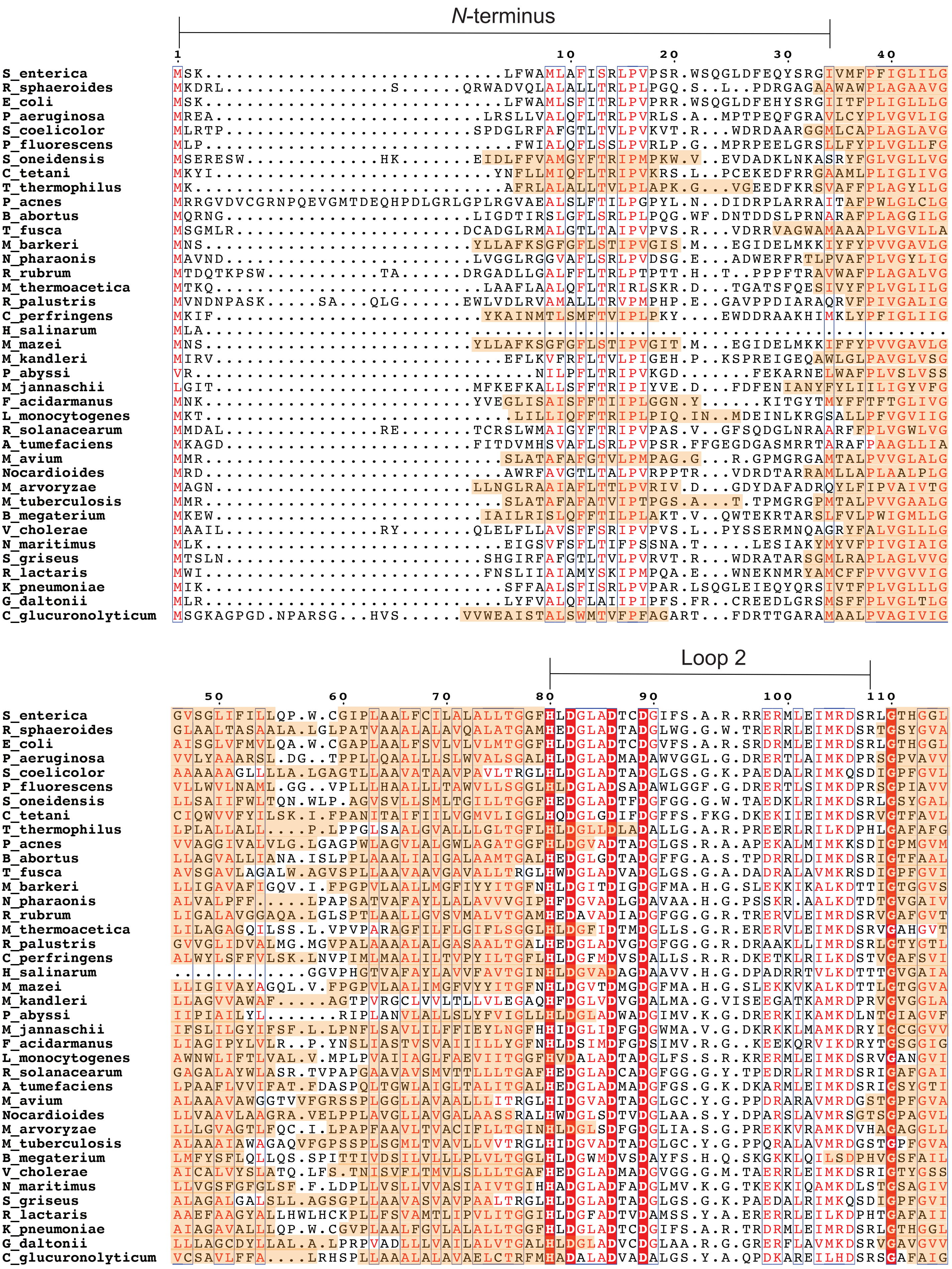
N-terminus
Loop 2
1
10
20
30
40
50
60
70
80
90
100
110
S_enterica
R_sphaeroides
E_coli
P_aeruginosa
S_coelicolor
P_fluorescens
S_oneidensis
C_tetani
T_thermophilus
P_acnes
B_abortus
T_fusca
M_barkeri
N_pharaonis
R_rubrum
M_thermoacetica
R_palustris
C_perfringens
H_salinarum
M_mazei
M_kandleri
P_abyssi
M_jannaschii
F_acidarmanus
L_monocytogenes
R_solanacearum
A_tumefaciens
M_avium
Nocardioides
M_arvoryzae
M_tuberculosis
B_megaterium
V_cholerae
N_maritimus
S_griseus
R_lactaris
K_pneumoniae
G_daltonii
C_glucuronolyticum

```
                                                                          Loop 4
                         120       130                 140       150       160
S_enterica           ALIFVLLAKILVVSELAL.RGT.P.........M...LAALA.AA.CAAGRGSAVLLMY.RH......RY.AREE
R_sphaeroides        ALVLVGLLRWSALAAALE.GG............V....ALLV.AA.AVLSRVPMVGLMA.LL......PN.ARGA
E_coli               ALIFVVLAKILVLSELAL.RGE.S.........I...LASLA.AA.CAVSRGTAALLMY.RH......RY.AREE
P_aeruginosa         VLVLVLLLKFSALAALLG.QGE...............AGLLP.LA.PWLARSSLPLLFL.TT......PY.ARPG
S_coelicolor         ALLFTLLAQVALAQAYD.GSW.AR.........G...ALAAV.VS.ATAARLALTTAARTGV......PA.ARPE
P_fluorescens        TLVLVLLLKFTALVALIE.QQQ...............GFALL.LA.PLIGRGALLGLFL.CT......PY.VRAG
S_oneidensis         ALIMVLLLKWQLLVELAL.YDP.VV........A...GSAMI.VA.HTVSRVVAASLIF.TE......TY.VRDD
C_tetani             ALIFDILIKYSALSFIIE.NNM..S.............YAII.IT.PIMSRCTLVFLFL.IG......KN.AKKN
T_thermophilus       VGGVYLLLLFQALALVQD...................PLFLL.LF.PGWARFAFLPFLH.RY......PL.L.GP
P_acnes              SIVLVLLVDAAAVVSLAG.TAG.SG........AWWRVAILVGLG.PALARAAILLATMTEV......PC.ARPG
B_abortus            TLVIWTGVKASLLMAIIA.RAGAGY........A...LLALI.GT.EAASRAGMLAFWH.AL......PS.ARPG
T_fusca              TLVFVVLLQVAAVAALAE.EGT.GA........A...VAGLA.AA.LVSGRLAITWACTRLV......GA.ARPE
M_barkeri            FCILLLLTLYGSIRAVQQ.EGS.AVFGSNLPVLM...FESMF.IA.EVSAKQSMLTIAA.FGKPIPPREK.QAYP
N_pharaonis          AVAVVFLGLVLAALALST.APL.AV........A....VTVV.IAAEVGAKLGMA.TVG.CL......GT.AGHE
R_rubrum             ALVLVLALRVAALAALAG.GPW...........A...MAALV.CA.AGLGRGAAVLMLG.LL......PP.ARTD
M_thermoacetica      AVITLLLLKFSLIFSLLS.LSP.GR.LWLAGMPL...SPFLL.LM.PVLARWAMVPAIT.CF......PY.ARRE
R_palustris          VLLVGFATRLSALASLPA.AM............A...IPALI.VA.HALGRAAIPVLAA.NM......PF.ARAD
C_perfringens        SVVLLLLVEFAGMFTVLN.KNL..D............MRILI.FI.PIASRVINGYFIV.SQ......EM.LGQS
H_salinarum          AVVVVVAGLVTGSLGVAALPT..WT........A....VGVV.VATEVGAKTSMA.AVA.CL......AH.APHD
M_mazei              FGILVLLAFYGSIRSVQE.EGI.AAFGSNLPFLM...FASMF.IA.EVSAKQSMLTIAA.FGKPLPRLKE.QTYP
M_kandleri           IGSMALLLAVASFGWIP....................FEVLV.PI.EVFSRFTVLPMAAVG.......EP.APAS
P_abyssi             AVVVVLFLQVYSLSMLPF....................YAIY.IA.ELNSKFSMLLGLA.TK.......K.PLGQ
M_jannaschii         FAIFFNLMAVISLSYILD...I.NI............LY.LL.VG.EVCAKLGMLSCST.FG.......N.PLIE
F_acidarmanus        MLFIIYIPAIAFLTYFKP...V.TG............FFIII.AG.EMASKYATLFSMY.RA.......K.AFGE
L_monocytogenes      AICFYFLFYGALFLSVPN.VQQ.I.........G...W.LFF.VL.PIVAKGMTMLLFA.KM.....TYAGSKE
R_solanacearum       AVCMALLLKWQLLTALAA.QHA.VA........V...MAAMV.AA.HAASRGVAVSYLL.TH......DY.VRME
A_tumefaciens        AMVLSFALRATALASLIE.TLPGKT........A...AACLI.AT.LVMSRALMVWHWQ.AL......PA.AKTS
M_avium              AVVVVVMAQALAFSALAA.GR..PV........P....VGVA.VA.VFAGRVAAVLACRRTV......PA.AAGS
Nocardioides         ATVVVAGVQAAALATLLD.QPL...........L...AGALV.....CLSRCALWIVCCTRV......PA.ARAD
M_arvoryzae          FMAMDLLFLFALAMTFAG.SPT.WL............FVPLL.VA.EGCAKVAQITIIA.FG.......K.SAHE
M_tuberculosis       AVVLVIALQGLAFATLTT.VG............I....AGIT.LA.VLSGRVTAVLVCRRLV......PA.AHGS
B_megaterium         SLMVLLLLRFSAIYELVS.LSS.LS............IWACL.IV.FTLPRIGAAFSLM.RD......KP.AKDT
V_cholerae           ALIMVLLGKYLLLTELAD.LTS...........L...VPVWL.LA.YTLSRAVAASLIR.NT......PY.VSDT
N_maritimus          GLVLYLVGLIITISLTNG...F.DL............FKAIL.IS.EILAKFSMVLMAS.LG.......N.SAAS
S_griseus            TLLLVLLAQVAVLFELYG.EGW.AH........G...AVGAV.VA.GTAARLALTQASRQGV......PA.ARPE
R_lactaris           GLCCYFLANVGIWSEIGE.KK................LLVAC.CI.FAFSRAMSGLAVV.SF......KA.AKNS
K_pneumoniae         ALIFVLVAKVLVIGELLL.RDT.H.........P...IAALA.AA.CAVGRGMAVLLMY.RH......RY.AREK
G_daltonii           GLVLALLLKYQALFAVTT.DK................WETLL.FF.PMVARFSQVQLTV.GS......KR.ARQD
C_glucuronolyticum   ALAMVYIVQFAAFASVPS.PW..FV........F....FSLW......AGRIAGQVPATGGF......AP.FSES

                      170       180            190                                 200
S_enterica           G..LGNVFIGKVSGR.QTC.IT.L.GLAV.I..V.ATV.......L.....LP.....G.M....QGL...AAMV
R_sphaeroides        G..LAQSLGRPDGRQ.AAL.AA.A.VGPG.V....ALL.......L.....AG.......P....AAL...VLPA
E_coli               G..LGNVFIGKIDGR.QTC.VT.L.GLAA.I..F.AAV.......L.....LP.....G.M....HGV...AAMV
P_aeruginosa         G..LGQAIAEHLPAR.SLP.WV.L.GVSF.G....LA........L.....AF.....G.L....AGL...LALL
S_coelicolor         G..LGAAVAGVVPAG.GAL.AA.T.AAVT.L..AGAAA.......GAYLGPYD.....A.L....RTA...LAVV
P_fluorescens        G..LGQALADHLPRV.AGR.QV.L.GLSV.L..A.CL........L.....LG.....G.Y....SGL...WAVL
S_oneidensis         ETSKSKPLAQHQGIN.DLF.IL.I.ASGV.L....VLL.......V.....LK.....G.I....AAL...SLLL
C_tetani             G..TGNLFIENVSVK.EFI.IS.F.IFMI.V.P..SVL.......L.....IG.....Y.K....YSV...IIIV
T_thermophilus       G..MAALVRGGPWPF......A.L.LPAL....P.FL........L...................LYP...LPAL
P_acnes              G..FGSLVAGVTTPR.SAA.IN.LTVLGC.V..C.ALA.......GLV...AG.....G.WMWCLVTV...SCAA
B_abortus            G..LADSMGQPQWET.VVC.GC.G.LGLA.L..L.AIG.......F.....LP.....S.G....GMVALINALV
T_fusca              G..LGAFVSGTVPYP.AAA.A..V.TCV..V..L.AVG.......L.....V......E.W....TWC...AAVA
M_barkeri            G..LGAMTINGATRK.NFL.IG.F.VFGA.I..V.CFL.......P.....FG.......W....IGL...LPYL
N_pharaonis          G..MGSQFTTVLDSE.MLV.VP.A.LAAV.P..A.AVA.......V............L.P....ATL...AAVL
R_rubrum             G..LGASFGRPQQAS.LAF.AL.I.LCLV.L....AVV.......L.....LP.....G.A....APA...VALV
M_thermoacetica      G..LGSLFGAGQGRK.ALL.LA.T.FSTM.I..L.SW........L.....TL.....G.Y....KGL...ILMF
R_palustris          G..LGKTAGRPAAAG.ALT.AV.V.LAVI.V....ALL.......C.....LP.....L.K....AAI...LALL
C_perfringens        S..LAKFFKEGTGKV.DEIILL.G.IYVL.V.AL.ITF.......F.....TL.....G.I....NYL...IAIL
H_salinarum          G..LGSQFTGNATPG.ALP.AV.A.GVAL.P..V.ALA.......S.....VP.....S.P....AAA...GALA
M_mazei              G..LGEMTINGATRK.NFL.IG.F.IFGA.V..V.CCL.......P.....FG.......L....IGL...IPYL
M_kandleri           Y..SGRVFTEYVDAD.QVL.LG.G.ILST.V..V.SL..........P...FS.....P.V....ATL...TCAV
P_abyssi             G..LGAYFMEGMNGR.QLA.IG.V.VLYV.L..L.YLP.......V.....VI.....Y.D....PSA...LFGV
M_jannaschii         G..TGRYFVKKADEK.FLT.IG.I.ILSL.P..L.LLI.......F.....SG....IERK....IVI...IAII
F_acidarmanus        G..LGKMFIDKVSAN.AIV.LN.V.I.PL.L..L.AFA.............FN.....LYN......I...AIIA
L_monocytogenes      G..LGSIFLGV.PWW.PIV.IA.Q.VIVL.A..A.LG........L.....FF.....S.Y....VGV...IAYV
R_solanacearum       G..KAKPVAQPMGWR.EAA.WA.A.VLGG.L....PLL.......W.....FG.....V.A....CAA...VAAI
A_tumefaciens        G..IAAGAGQPGESD.RNI.AL.V.TGLL.V..F.ILF.......T.....LH.....A.L....PILSIALVMA
M_avium              S..LGAAVAGSQPAT.VAA.AW.V.AVLL.G..M.SLV.......AG....PR.....P.W....HGP...VAVL
Nocardioides         G..LGADVARTVPLP.VAV.L............................................GGL...LLSA
M_arvoryzae          G..MGSYMIARMKKE.HYL.AA.V.IGAWIA..I.GIAIIGAAIIP.....GGGNPLRVIM....AGG...LAML
M_tuberculosis       T..LGSRVAGTQPAP.VVA.AW.L.AVLL.A..V.SVP.......AG....PR.....P.W....QGP...IAVL
B_megaterium         G..LAAYFQQGVTKR.SIY.GF.I.IMSL.F.LV.AIF.......V.....IF.......M....DNK...FIIL
V_cholerae           DSSKSKPLAQQLSGT.DVA.VL.S.LTAL.A....TLL.......Y.....FS.....W.Q....FIG...VMIA
N_maritimus          G..SNSPFVHLMKDKRKLV.AA.F.IIM..L..I.PVA.......L.....IG.....E.T....TGL...IMLG
S_griseus            G..LGAVVASTVPAG.RAL.LA.A.VVTV.A..LCAAA.......GAPLGPYG.....A.L....RHA...LAAL
R_lactaris           G..LLRTFQDGAAKR.RVR.VVMM.LWAV.LTAF.ILL.......K.....IS.....P.A....AGT...AALV
K_pneumoniae         G..LGNLFIGKVSLQ.QTL.VT.M.AMGV.A..L.AT........V.....LL.....G.L....QGL...RAAL
G_daltonii           G..LGSLFIGGAGSM.QVA.VA.A.FFTV.V..T.GW........L.....LL.....G.L....PGI...GCAA
C_glucuronolyticum   G..FGGLIIGTVHWW.WIA.VW.W.AILS.G..F.SLA.......V.....A......G.P....WIA...LASA
```

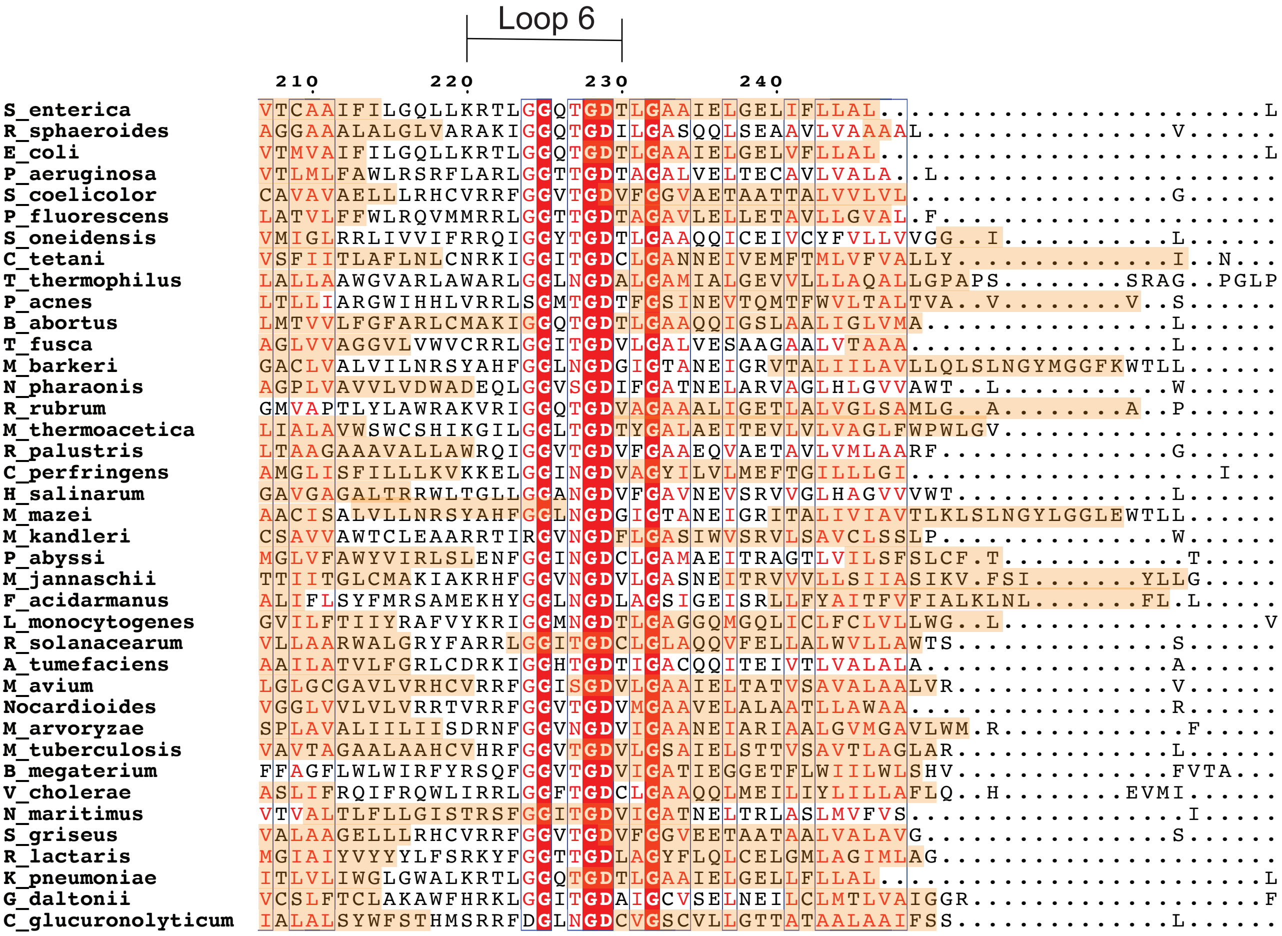
Loop 6
210
220
230
240
S_enterica
R_sphaeroides
E_coli
P_aeruginosa
S_coelicolor
P_fluorescens
S_oneidensis
C_tetani
T_thermophilus
P_acnes
B_abortus
T_fusca
M_barkeri
N_pharaonis
R_rubrum
M_thermoacetica
R_palustris
C_perfringens
H_salinarum
M_mazei
M_kandleri
P_abyssi
M_jannaschii
F_acidarmanus
L_monocytogenes
R_solanacearum
A_tumefaciens
M_avium
Nocardioides
M_arvoryzae
M_tuberculosis
B_megaterium
V_cholerae
N_maritimus
S_griseus
R_lactaris
K_pneumoniae
G_daltonii
C_glucuronolyticum
