# Supplementary material for: Insights into the Relationship between Cobamide Synthase and the Cell Membrane
Source: mBio. 2021 Mar 23;12(2):e00215-21. doi: 10.1128/mBio.00215-21 (PMC8092220; doi:10.1128/mBio.00215-21)
Supplement: TABLE S1 [file mBio.00215-21-st001.pdf]

| Table S1. Strains and plasmids used in this study | | |
|---|---|---|
| **Strains** | **Genotype** | **Reference or Source** |
| JE6583 | *metE205 ara-9* | Laboratory collection |
| Derivatives of strain JE6583 | | |
| JE22070 | / pCV1 | Laboratory collection |
| JE15792 | / pBAD33 | Laboratory collection |
| JE8248 | Δ*cobS1313* | Laboratory collection |
| JE15888 | Δ*cobS1313* / pCOBS69 | |
| JE15762 | Δ*cobS1313* / pCOBS68 | |
| JE15800 | Δ*cobS1313* / pCOBS71 | |
| JE15802 | Δ*cobS1313* / pCOBS73 | |
| JE15937 | Δ*cobS1313* / pCOBS79 | |
| JE16281 | Δ*cobS1313* / pCOBS82 | |
| JE16282 | Δ*cobS1313* / pCOBS83 | |
| JE16283 | Δ*cobS1313* / pCOBS84 | |
| JE16284 | Δ*cobS1313* / pCOBS85 | |
| JE16523 | Δ*cobS1313* / pCOBS89 | |
| JE25590 | Δ*cobS1313* / pCOBS104 | |
| JE25591 | Δ*cobS1313* / pCOBS105 | |
| JE25592 | Δ*cobS1313* / pCOBS106 | |
| JE25593 | Δ*cobS1313* / pCOBS107 | |
| JE25594 | Δ*cobS1313* / pCOBS108 | |
| JE25595 | Δ*cobS1313* / pCOBS109 | |
| JE25839 | Δ*cobS1313* / pCOBS78 | |
| JE7088 | Δ*metE2702 ara-9* | Laboratory collection |
| **Derivatives of strain JE7088** | | |
| JE11685 | / pBAD24 | Laboratory collection |

| | | |
|---|---|---|
| JE22263 | / pCV1 | Laboratory collection |
| ***Escherichia coli strains*** | | |
| JE6663 C41(λDE3) | F – *ompT hsdSB (rB- mB-) gal dcm* (λDE3) | Avidis |
| **Plasmid** | **Genotype** | **Source** |
| pTEV5 | Overexpression vector that fuses the *N* terminus of the protein of interest to a $H_6$ tag, which can be removed by rTEV protease, *bla*$^+$ | (20) |
| pBAD24 | complementation vector, $P_{araBAD}$, *bla*$^+$ | (21) |
| pBAD33 | complementation vector, $P_{araBAD}$, *cat*$^+$ | |
| pCOBS68 | *S.* Typhimurium *cobS*$^+$ pBAD33-SD1 | |
| pCOBS69 | *S.* Typhimurium *cobS*$^+$ pBAD24 | |
| pCOBS71 | *S.* Typhimurium *cobS* H80A pBAD33-SD1 | |
| pCOBS73 | *S.* Typhimurium *cobS* M104A pBAD33-SD1 | |
| pCOBS78 | *S.* Typhimurium *cobS* D89A pBAD33-SD1 | |
| pCOBS79 | *S.* Typhimurium *cobS* D86A pBAD33-SD1 | |
| pCOBS82 | *S.* Typhimurium *cobS* G45E pBAD33-SD1 | |
| pCOBS83 | *S.* Typhimurium *cobS* D229A pBAD33-SD1 | |
| pCOBS84 | *S.* Typhimurium *cobS* G232A pBAD33-SD1 | |
| pCOBS85 | *S.* Typhimurium *cobS* G225A pBAD33-SD1 | |
| pCOBS89 | *S.* Typhimurium *cobS* D82A pBAD33-SD1 | |
| pCOBS104 | *S.* Typhimurium *cobS* L168V pBAD24 | |
| pCOBS105 | *S.* Typhimurium *cobS* R105K pBAD24 | |
| pCOBS106 | *S.* Typhimurium *cobS* R164K pBAD24 | |
| pCOBS107 | *S.* Typhimurium *cobS* R108K pBAD24 | |
| pCOBS108 | *S.* Typhimurium *cobS* R159K pBAD24 | |
| pCOBS109 | *S.* Typhimurium *cobS* R108E pBAD24 | |
| pCOBS5 | *S.* Typhimurium *cobS* pET15b | (11) |
