# Supplementary material for: Insights into the Relationship between Cobamide Synthase and the Cell Membrane
Source: mBio. 2021 Mar 23;12(2):e00215-21. doi: 10.1128/mBio.00215-21 (PMC8092220; doi:10.1128/mBio.00215-21)
Supplement: TABLE S2 [file mBio.00215-21-st002.pdf]

Table 2. Primers used in this work.	
**Primer Name**	**Primer Sequence (5'-3')**
cobS_G45E	GGGTTGATTCTGGAGGGCGTAAGCGGC
cobS_H80A	GCCCATCCAGGGCAAAACCACCGGTCAGCAGCG
cobS_D86A	GATGGGCTGGCCGCTACCTGCGATGGC
cobS_D89A	TGGCCGATACCTGCGCTGGCATTTTTTCCGC
cobS_M104A	GCCGTGAGCGAATGCTGGAGATTGCGCGTGATAGTCG
cobS_D82A	GTGGTTTTCACCTGGCTGGGCTGGCCGATAC
cobS_D229A	GTCAAACCGGCGCTACGCTGGGCGC
cobS_G232A	CGATACGCTGGCCGCGGCGATCG
cobS_G225A	CGTACGCTTGGCGCTCAAACCGGCGAT
cobS_L168V 1	CGCCCGTGAAGAGGGGGTCGGCAATGTATTTATC
cobS_L168V 2	GATAAATACATTGCCGACCCCCTCTTCACGGGCG
cobS_R159K 1	CCGTTTTATTGATGTACAAGCATCGCTACGCCCGTG
cobS_R159K 2	CACGGGCGTAGCGATGCTTGTACATCAATAAAACGG
cobS_R164K 1	CGTCATCGCTACGCCAAAGAAGAGGGGCTTGG
cobS_R164K 2	CCAAGCCCCTCTTCTTTGGCGTAGCGATGACG
cobS_R105K 1	CGAATGCTGGAGATTATGAAGGATAGTCGTCTGGGAAC
cobS_R105K 2	GTTCCCAGACGACTATCCTTCATAATCTCCAGCATTCG
cobS_R105E 1	GAATGCTGGAGATTATGGAGGATAGTCGTCTGGGAAC
cobS_R105E 2	GTTCCCAGACGACTATCCTCCATAATCTCCAGCATTC
cobS_R108K 1	CGAATGCTGGAGATTATGAAGGATAGTCGTCTGGGAAC
cobS_R108K 2	GTTCCCAGACGACTATCCTTCATAATCTCCAGCATTCG
